# Design of a 5G millimeter-wave wideband high-gain metamaterial-based antenna

**DOI:** 10.1038/s41598-026-55066-y

**Published:** 2026-06-04

**Authors:** Islam Osama, Mohamed Elhefnawy, Ahmed Yahya

**Affiliations:** 1 Department of Electrical Engineering, Faculty of Engineering, Al-Azhar University, Nasr City, Cairo Egypt; 2 Department of Electrical Engineering, Faculty of Engineering, October 6 University, 6th, October City, Giza Egypt

**Keywords:** Equivalent circuit model, Characteristic mode analysis (CMA), Millimeter-wave, Metamaterial, Metasurface, Double Negative, 5G, Engineering, Materials science, Physics

## Abstract

This research paper presents a novel high-gain, wideband metamaterial (MTM)-based antenna designed for 5G millimeter-wave (mm-wave) applications. The antenna features a dual-band H-shaped patch radiator printed on a 15 mm × 15 mm Rogers 5880 substrate, backed by a metallic ground plane with a square aperture. A 2 × 2 MTM superstrate layer is positioned 6.1 mm above the patch antenna to improve gain and bandwidth. The MTM layer, fabricated on a 0.2 mm Rogers 4003 C substrate, incorporates H-shaped metallic patterns on the top surface and circular resonators on the bottom. Simulated effective constitutive parameters confirm negative permittivity ($\varepsilon$), negative permeability ($\mu$), and negative refractive index (*n*) within the target frequency range, validating the metamaterial properties. The S-parameters derived from the equivalent circuit model developed in ADS closely match those obtained from the CST simulations. Characteristic Mode Analysis (CMA) is conducted to examine the resonance behavior of the proposed MTM-based antenna. The experimental results demonstrate the wideband and high-gain performance of the proposed antenna, featuring an impedance bandwidth of 4.8 GHz, peak gains of 11.4 dBi at 30 GHz and 9.19 dBi at 27.4 GHz, and a radiation efficiency of 95% across the entire operating frequency band.

## Introduction

 Millimeter-wave technology has emerged to address limitations such as low data rates and spectrum congestion. To enable high-frequency wireless communication, wideband, high-gain, compact, and low-cost antennas are essential. Although millimeter-wave systems offer high data rates and low communication delay, they suffer from severe path loss and environmental factors that limit coverage. This challenge can be mitigated by using high-gain, directive antennas, which can be realized through various techniques such as multiple-input multiple-output (MIMO), metamaterials, electromagnetic band-gap (EBG) structures, dielectric superstrates, and substrate-integrated waveguide (SIW) designs^1,2^.

Metamaterials provide an effective solution for enhancing antenna performance parameters, including gain, directivity, bandwidth, and efficiency. Owing to their ability to manipulate electromagnetic waves, they are commonly integrated with antennas and other microwave devices to improve performance. A defining characteristic of metamaterials is their capability to exhibit a negative refractive index, enabled by their negative ($\varepsilon$) and ($\mu$) properties. Over the past decade, research in communication systems has significantly increased, with particular emphasis on advancements in antenna technologies. Metasurface (MS)-based antennas have recently attracted significant attention because of their compact structures, high gain, wide bandwidth, and cost-effectiveness^3–8^. A mm-wave slot antenna with FSS reflectors is proposed for 5G applications, achieving a gain of 10.3 dBi at 28 GHz, which is 5.3 dBi higher than the case without FSS. The antenna also exhibits a broad bandwidth from 25.5 to 30.8 GHz, and a radiation efficiency of 90.5%. However, the dielectric substrate and FSS conductors could potentially reduce the overall efficiency^9^. Paper^10^ presents a MTM-enhanced bowtie antenna for 5G microwave applications, achieving a significant bandwidth increase from 32% to 55% and a gain improvement from 6.25 dBi to 8.56 dBi at 28 GHz. This approach enhances performance without increasing size or impacting radiation characteristics, making it suitable for compact designs. Similarly^11^ reports a compact antenna design using a single-negative MTM unit cell, meander line, and defected ground structure (DGS). This design achieves a 26.6% fractional bandwidth (3.26–4.26 GHz) and a peak gain of 1.26 dBi, suitable for 5G and IoT. In^12^, the authors present a tunable single-layer MTM unit cell designed for 5G applications, exhibiting epsilon-negative (ENG) behavior and a near-zero refractive index within the 28–32 GHz frequency range. Reference^13^ introduces a MS-based microstrip patch antenna with enhanced gain, wide impedance bandwidth, and broad axial ratio bandwidth, suitable for WLAN and Wi-Fi applications. A compact, low-profile MS-based circularly polarized (CP) microstrip patch antenna featuring a slotted patch and a 4 × 4 metasurface array is proposed in^14^. This design achieves a gain of 6.3 dBic at 6.25 GHz, an impedance bandwidth of 5.45–7.55 GHz, and an axial ratio bandwidth of 6.02–6.35 GHz, making it ideal for satellite and WLAN applications. The study in^15^ demonstrated a remarkable enhancement in antenna gain over a broad bandwidth for 5G applications using a compact, single-layer MTM lens. In^16^, machine learning was applied to design a graded-index metasurface lens, significantly improving antenna gain within the 5G N261 band (27.5–28.35 GHz) for mobile communications. Reference^17^ developed a low-profile, high-gain, wideband CP antenna with a MS superstrate for 5G mmWave systems. The compact antenna in^18^, based on a zero-index metasurface, achieved enhanced gain and circular polarization with an operational frequency range from 24.6 to 31.8 GHz. A CPW-fed ultra-wideband strawberry-shaped printed monopole antenna coupled with a single-layer FSS reflector was proposed in^19^, achieving an average gain of 9.68 dBi across 3.05–11.9 GHz, suitable for UWB and ground-penetrating radar applications. The FSS array notably increased gain from 1.65 dBi to 7.87 dBi at lower frequencies and from 6.3 dBi to 9.68 dBi at higher frequencies. Moreover^20^ introduced a 16-port massive MIMO antenna system employing double-negative (DNG) and epsilon-negative (ENG) metamaterials, achieving up to 20 dBi gain and 99% efficiency in the 25.5–29 GHz band with isolation above 25 dB. The integration of MTMs significantly improves performance, providing a 5 dB improvement in isolation and a 1.9 dB gain increase compared to conventional designs. The authors in^21^ presented a dual-band MS-based antenna designed for 5G applications. By integrating an MS layer above a microstrip patch antenna, the design achieves dual-band functionality with improved directivity and efficiency compared to conventional antennas. The antenna operates at 26.4 GHz and 27.6 GHz, achieving total efficiencies of 91.2% and gains of 10 dBi and 9.6 dBi, respectively.

This work contributes to the development of efficient and compact communication systems for next-generation wireless networks by advancing both theoretical understanding and practical implementation of double-negative (DNG) metamaterial structures. Although extensive research has been conducted on DNG metamaterial-based antennas, this work introduces a novel design that significantly improves the overall performance trade-off in terms of gain, bandwidth, radiation efficiency, and compactness. Specifically, the proposed antenna introduces a modified H-shaped patch configuration with a defected ground structure (DGS), together with a newly designed DNG metamaterial unit cell structure. The integration of this engineered MTM layer above the dual-band patch antenna enables multi-mode excitation and broadband operation in the mm-wave frequency range targeted for 5G systems. The proposed antenna achieves a measured peak gain of 11.4 dBi, a fractional bandwidth of 16.6%, and a radiation efficiency of 95%. Compared with a previously published mm-wave antenna design, the proposed antenna provides a more balanced overall performance, simultaneously achieving high gain, wide bandwidth, and high efficiency in a compact structure.

The proposed MTM-superstrate antenna offers a simpler and more compact design compared to beam-scanning and reflectarray solutions, as it does not require complex feeding networks or active components. While it does not provide beam-steering capability, it enables high gain with low complexity, making it suitable for compact and cost-effective integration in 5G applications^22,23^.

This paper is organized as follows: Sect.  2 presents the patch antenna configuration and corresponding simulation results. Section  3 provides details on the configuration of the metamaterial unit cells. Section  4 discusses the integrated antenna-metamaterial system and its simulation outcomes. The CMA of the proposed metamaterial-based antenna is provided in Sect.  5. Finally, Sect.  6 covers the fabrication process and the experimental measurement results.

### Patch antenna configuration

**Fig. 1:**
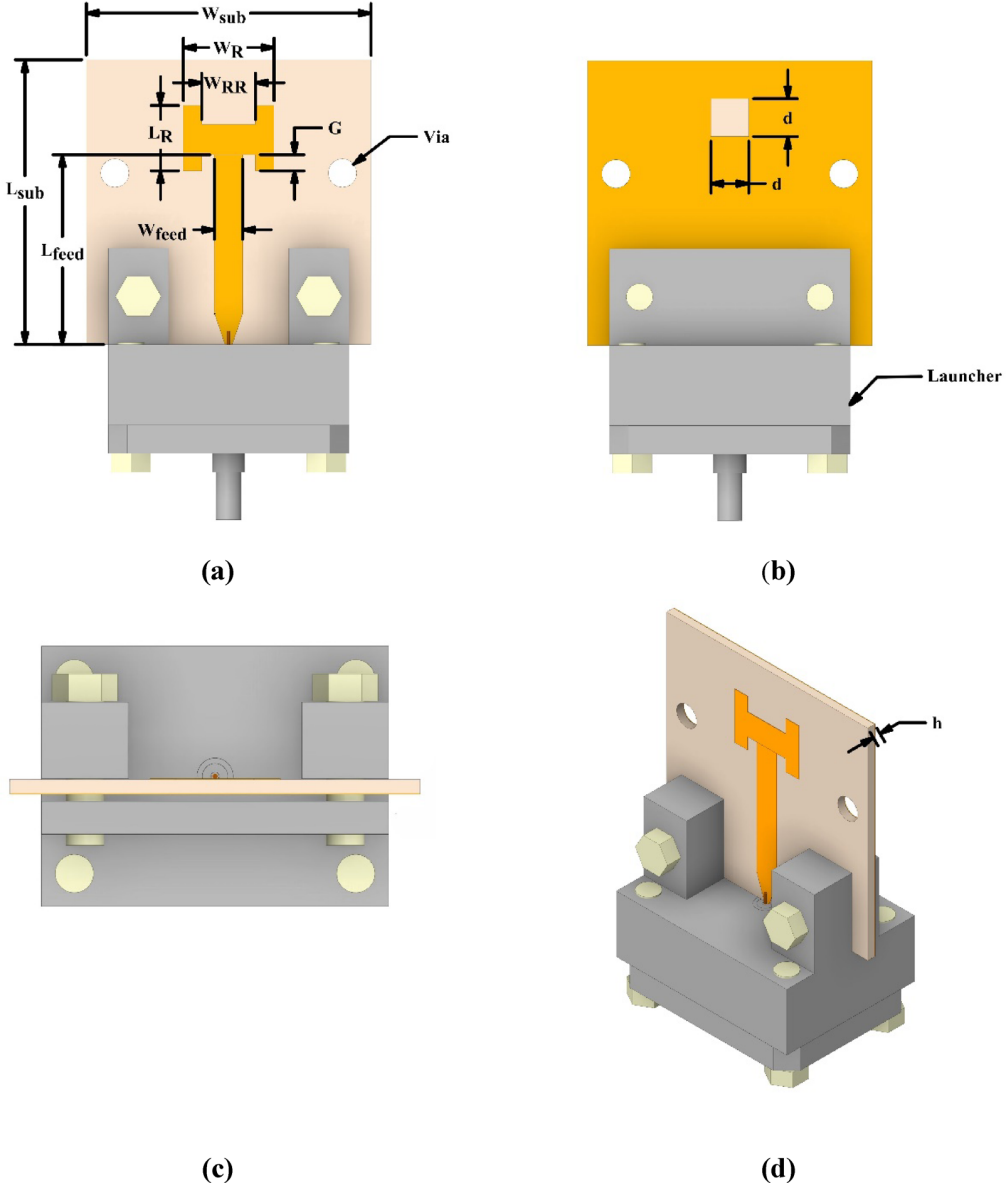
Patch antenna layout: (**a**) top view, (**b**) bottom view, (**c**) side perspective, and (**d**) 3D view.

The structural layout of the patch antenna, illustrated in Figure. 1, is realized on a Rogers-Duroid™ 5880 substrate. This material was selected specifically for its low dielectric constant ($\:{\boldsymbol{\epsilon\:}}_{\boldsymbol{r}}$ **= 2.2**) and extremely low loss tangent ($\:\mathrm{t}\mathrm{a}\mathrm{n}\delta\:=0.0009$). A low dielectric constant reduces field confinement, thereby minimizing dielectric losses and improving radiation efficiency. Moreover, substrates with lower permittivity store less reactive energy, leading to a reduced quality factor and suppressed surface-wave effects; consequently, the impedance bandwidth is enhanced. The substrate thickness of **h** = 0.508 mm was chosen to further mitigate surface-wave excitation and dielectric losses, resulting in improved radiation efficiency and stable gain performance. The primary patch element is designed for impedance matching to a 50 Ω load^24^. To emulate the measurement setup, the antenna is terminated with a launcher connector in the simulation, as shown in Figure. 1. The design methodology comprises three distinct phases: initial design based on mathematical modeling, impedance matching enhancement between the feed-line and radiating patch to improve return loss at the resonant frequency, and final optimization for miniaturization, resonance adjustment, and further enhancement of return loss and antenna gain. This subsection briefly reviews the mathematical models used to determine the initial dimensions of the rectangular microstrip patch antenna^24^. The design begins by calculating the patch width (${W_R}$) using Eq. (1).

1 $$W_{R} = \frac{C}{{02F_{r} }}\sqrt {\frac{2}{{\varepsilon _{r} + 1}}}$$

The substrate relative permittivity is denoted by $\varepsilon {}_{r}$, *C *represents the speed of light, and ${F_R}$ is the desired center frequency. The radiating patch length is determined through a two-step process, starting with the calculation of the effective permittivity (${\varepsilon _{eff}}$) as:

2 $$\varepsilon _{{eff}} = \frac{{\varepsilon {}_{r} + 1}}{2} + \frac{{\varepsilon _{r} - 1}}{{2\sqrt {1 + 12\frac{h}{{W_{R} }}} }}.$$

where ***h*** represents the substrate thickness. Subsequently, the excess length (ΔL) caused by the fringing fields is obtained using Eq. (3)

3 $$\Delta L = 0.42h\frac{{\left( {\varepsilon _{{eff}} + 0.3} \right)\left( {\tfrac{{W_{R} }}{h} + 0.264} \right)}}{{\left( {\varepsilon _{{eff}} - 0.258} \right)\left( {\tfrac{{W_{R} }}{h} + 0.8} \right)}}.$$

The actual length (${L_R}$) is then determined using the following equation:

4 $$L_{R} = \frac{C}{{2F_{r} \sqrt \varepsilon _{{eff}} }} - 2\Delta L.$$

After determining the initial dimensions of the radiating patch, the dimensions of the dielectric substrate including its width (${W_{Sub}}$) and length (${L_{Sub}}$), are calculated utilizing the following equations:

5 $$W_{{Sub}} = W_{R} + 6h.$$

6 $$L_{{Sub}} = L_{R} + 6h.$$

Next, the inset feed gap is determined using the relation provided in^25^.

7 $$G = \frac{{4.65 \times 10^{{ - 18}} C.F_{r} }}{{\sqrt {2\varepsilon _{{eff}} } }}.$$

Finally, the microstrip feed-line width and length are designed to obtain a characteristic impedance of 50 Ω using Eqs. (8) and (9), respectively:

8 $$W_{{feed}} = \frac{2}{\pi }\left\{ {b - 1 - \ln \left( {2b - 1} \right) + \frac{{\varepsilon _{r} - 1}}{{2\varepsilon _{r} }}\left[ {\ln \left( {b - 1} \right) + 0.39 - \frac{{0.61}}{{\varepsilon _{r} }}} \right]} \right\}.$$

9 $$L_{{feed}} = 3h.$$

Where

10 $$b = \frac{{60\pi ^{2} }}{{Z_{C} \sqrt {\varepsilon _{r} } }}.$$

It should be noted that the final design parameters were determined by optimization and tuning using CST MWS simulation software, and the results are listed in Table 1.

**Table 1:** Geometric parameters of the optimized patch antenna.

parameter	$\:{\boldsymbol{W}}_{\boldsymbol{S}\boldsymbol{u}\boldsymbol{b}}$	$\:{\boldsymbol{L}}_{\boldsymbol{S}\boldsymbol{u}\boldsymbol{b}}$	$\:{\boldsymbol{W}}_{\boldsymbol{R}}$	$\:{\boldsymbol{L}}_{\boldsymbol{R}}$	$\:{\boldsymbol{W}}_{\boldsymbol{f}\boldsymbol{e}\boldsymbol{e}\boldsymbol{d}}$	$\:{\boldsymbol{L}}_{\boldsymbol{f}\boldsymbol{e}\boldsymbol{e}\boldsymbol{d}}$	$\:{\boldsymbol{W}}_{\boldsymbol{R}\boldsymbol{R}}$	h	d	G
Value (mm)	15	15	4.78	3.45	1.49	10	2.84	0.508	2	0.85

**Fig. 2:**
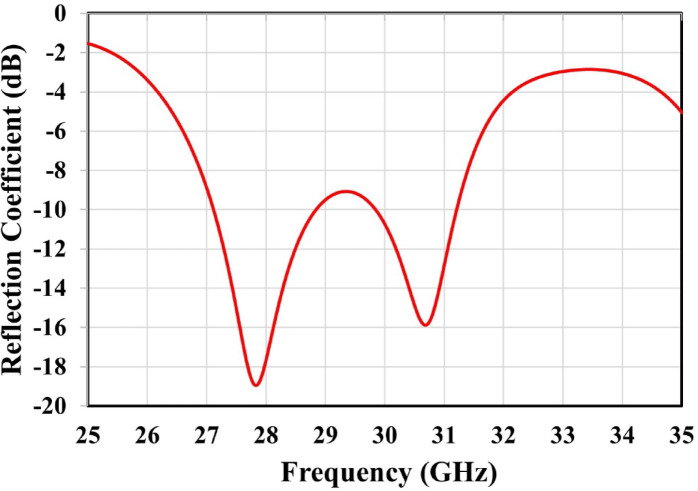
Simulated reflection coefficient of the proposed patch antenna.

**Fig. 3:**
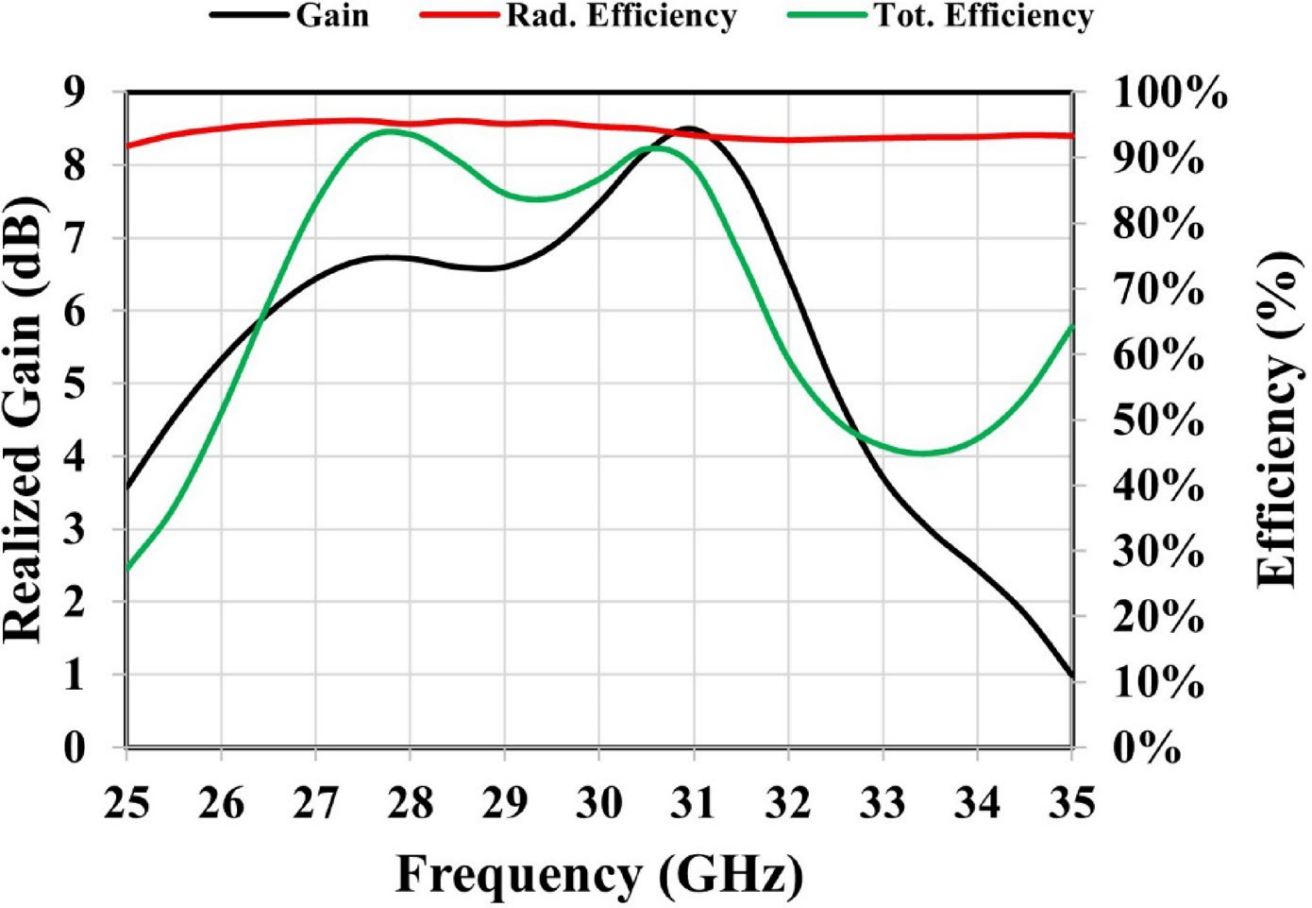
Simulated realized gain and efficiency of the proposed patch antenna.

The proposed antenna demonstrates dual resonances at 27.85 GHz and 30.72 GHz, exhibiting reflection coefficients of − 19.50 dB and − 16 dB, respectively, as shown in Fig. 2. The S11 parameter was obtained by exciting the antenna through the launcher connector. The corresponding impedance bandwidths are 1.67 GHz and 1.32 GHz, resulting in relative bandwidths of 6% and 4.29%, respectively. CST simulation results for realized gain, total efficiency, and radiation efficiency are illustrated in Fig. 3. At 27.85 GHz, the realized gain is approximately 6.7 dBi, increasing to 8.1 dBi at 30.72 GHz. The antenna demonstrates an efficiency of approximately 94%.

### Metamaterial unit-cell configuration

**Fig. 4:**
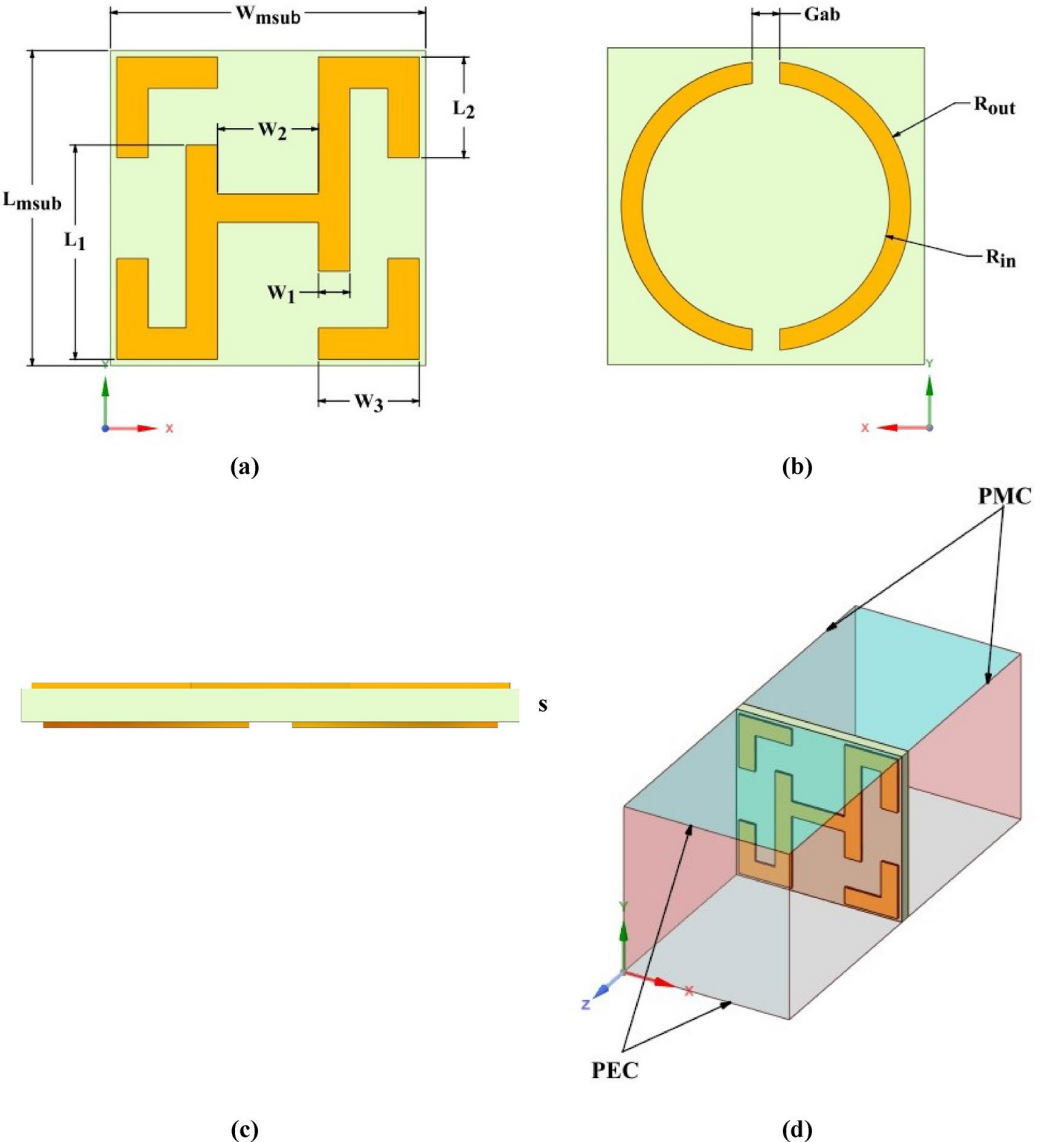
Unit cell layout: (**a**) top view, (**b**) bottom view, (**c**) side perspective, and (**d**) 3D perspective with boundary conditions.

The proposed MTM unit cell consists of a novel H-shaped structure on the top layer and a slotted circular ring on the bottom layer, both structures are fabricated on a single-layer Rogers Duroid™ 4003 C substrate with a thickness **s** = 0.2, **ε**_**r**_ = 3.38, and loss tangent **tanδ** = 0.0027. The configuration of the MTM unit cell is illustrated in Fig. 4, while the corresponding MTM parameter values are presented in Table 2.

The effective constitutive parameters, including ($\varepsilon$), ($\mu$), and (n), are obtained from the extracted simulated S-parameters, as explained below^26,27^.

**Table 2:** The optimized geometrical parameters for the unit cell.

parameter	$\:{\boldsymbol{W}}_{\boldsymbol{m}\boldsymbol{S}\boldsymbol{u}\boldsymbol{b}}$	$\:{\boldsymbol{L}}_{\boldsymbol{m}\boldsymbol{S}\boldsymbol{u}\boldsymbol{b}}$	$\:{\boldsymbol{W}}_{1}$	$\:{\boldsymbol{W}}_{2}$	$\:{\boldsymbol{W}}_{3}$	$\:{\boldsymbol{L}}_{1}$	$\:{\boldsymbol{L}}_{2}$	$\:{\boldsymbol{R}}_{\boldsymbol{o}\boldsymbol{u}\boldsymbol{t}}$	$\:{\boldsymbol{R}}_{\boldsymbol{i}\boldsymbol{n}}$	s	Gab
Value (mm)	3	3	0.30	0.96	0.96	2.04	0.96	1.37	1.17	0.2	0.26

**Fig. 5:**
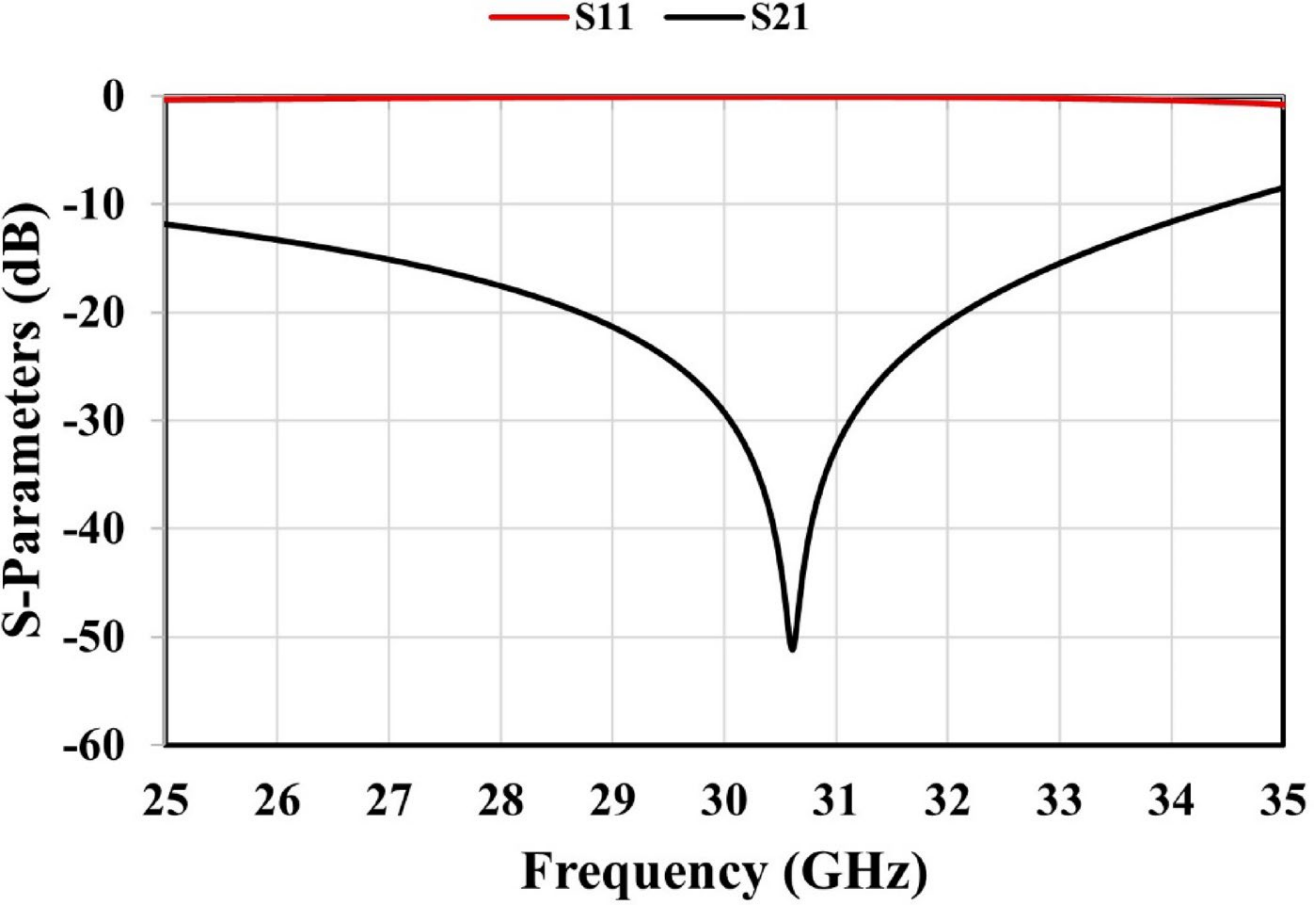
Simulated S-parameters of the proposed unit cell.

To obtain the S-parameters, the proposed unit cell with thickness **s** was simulated utilizing the CST full-wave electromagnetic solver, as depicted in Fig. 4(d). Perfect Magnetic Conductor (PMC) boundary conditions were applied to the faces parallel to the YZ-plane, while Perfect Electric Conductor (PEC) boundary conditions were imposed on the faces parallel to the XZ-plane. A linearly polarized Transverse Electromagnetic (TEM) wave was launched from waveguide port 1 to evaluate the reflection and transmission coefficients at both ports^28^. As illustrated in Fig. 5, the S-parameter results indicate that the unit cell exhibits a high transmission magnitude over a wide bandwidth. The obtained simulated reflection and transmission coefficients are related to the refractive index *n* and impedance z, as indicated in Eqs. (11) and (12), respectively, where $\:{R}_{01}=(z-1)/(z+1)$^26,27^. The impedance z is then obtained by solving Eqs. (11) and (12), after that the refractive index n can be determined from Eq. (16). Finally, the effective permittivity ($\varepsilon$) and permeability ($\mu$) of the MTM are calculated using Eqs. (17) and (18).

11 $$S_{{11}} = \frac{{R_{{01}} \left( {1 - e^{{i2nk_{0} s}} } \right)}}{{1 - R_{{01}}^{2} e^{{i2nk_{0} s}} }}.$$

12 $${S_{21}}=\frac{{\left( {1 - R_{{01}}^{2}} \right){e^{i2n{k_0}s}}}}{{1 - R_{{01}}^{2}{e^{i2n{k_0}s}}}}.$$

13 $$z = \pm \sqrt {\frac{{\left( {1 + S_{{11}} } \right)^{2} - S_{{21}}^{2} }}{{\left( {1 - S_{{11}} } \right)^{2} - S_{{21}}^{2} }}} .$$

14 $$n_{1} \sin (\varphi _{1} ) = n_{2} \sin (\varphi _{2} ).$$

15 $$e^{{ink_{0} d}} = \frac{{S_{{21}} }}{{1 - S_{{11}} \frac{{z - 1}}{{z + 1}}}}.$$

16 $$n = \frac{1}{{k_{0} d}}\left[ {\left\{ {\left[ {\ln \left( {e^{{ink_{0} d}} } \right)} \right]^{{\prime \prime }} + 2m\pi } \right\} - i\left[ {\ln \left( {e^{{ink_{0} d}} } \right)} \right]^{\prime } } \right].$$

17 $$\varepsilon =\frac{n}{z}.$$

18 $$\mu =nz.$$

Where ${\left( - \right)^\prime }$ refer to the real component, ${\left( - \right)^{\prime \prime }}$ refer to the imaginary component, ${k_0}$ is the wave number.

**Fig. 6:**
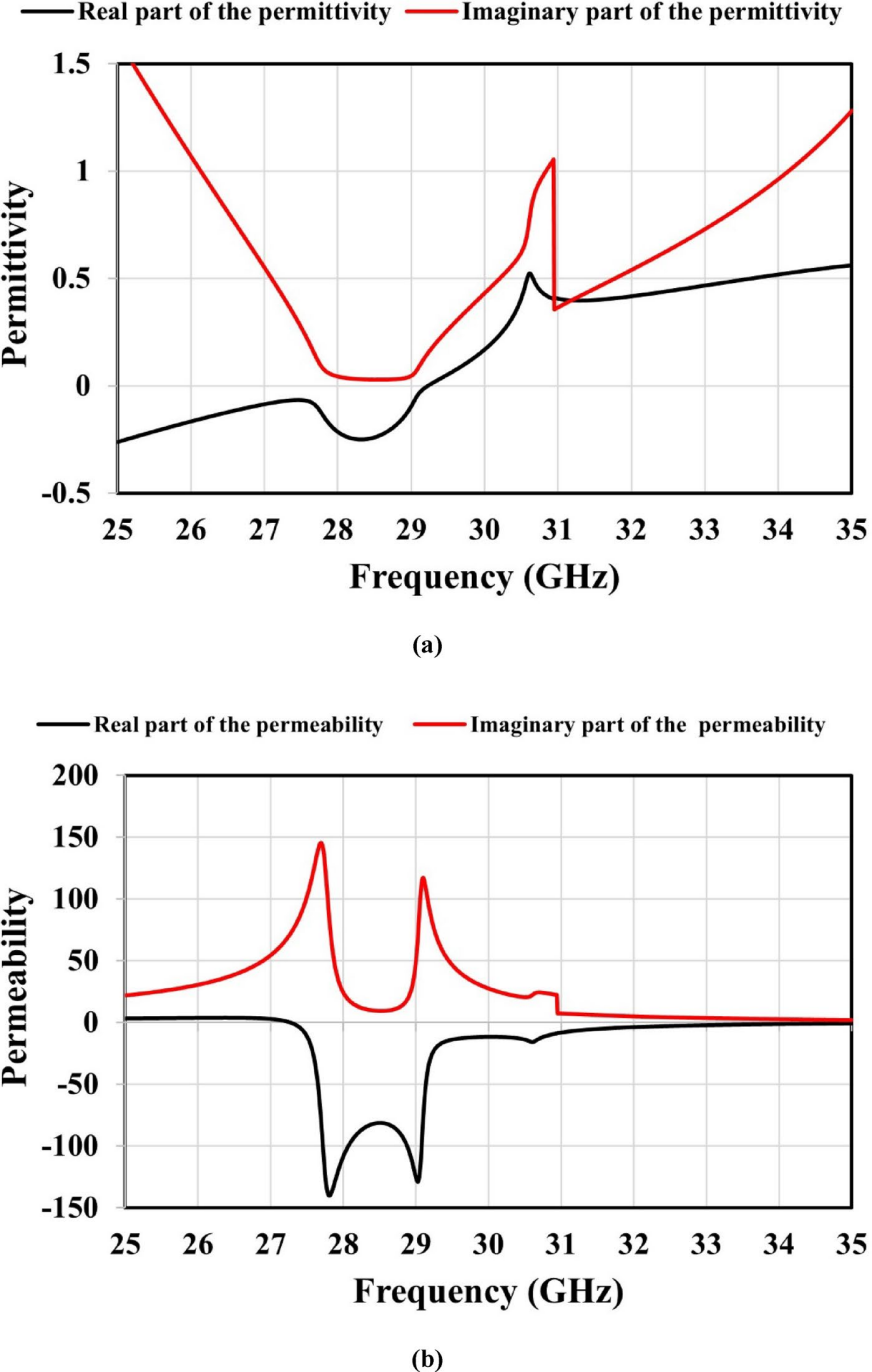

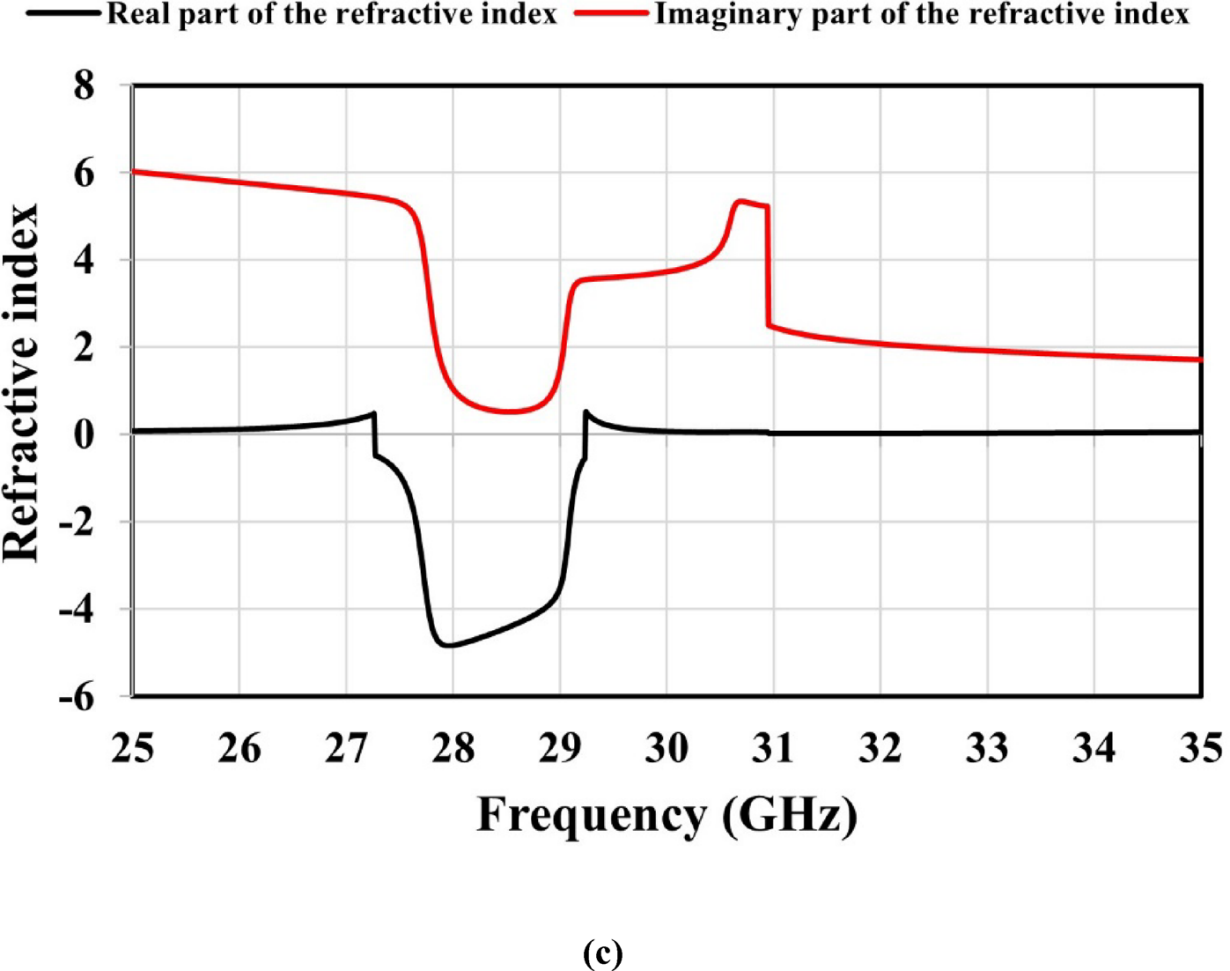
Simulated results for the unit cell’s effective constitutive parameters: (a) permittivity, (b) permeability, and (c) refractive index.

A metamaterial exhibits a negative (n) when both its effective ($\varepsilon$) and ($\mu$) are negative within a specific frequency range, resulting in electromagnetic wave propagation that fundamentally differs from that in conventional positive-index materials. The phase velocity is affected by the real part of the refractive index, whereas the propagation losses and antenna efficiency are affected by the imaginary part.

Snell’s law, expressed in Eq. (14), governs wave transmission across media with different refractive indices^29^. As shown in Fig. 6, both the ($\varepsilon$) and ($\mu$) real parts are negative between 27.3 and 29.2 GHz, confirming DNG behavior and a corresponding negative refractive index. Additionally, the imaginary parts of these parameters are positive, remain low, and achieve minimum losses within this frequency range. These characteristics of the constitutive parameters improve the focusing of incident waves and enhance the antenna’s gain and efficiency.

### Simulation results of the metamaterial-based antenna

**Fig. 7:**
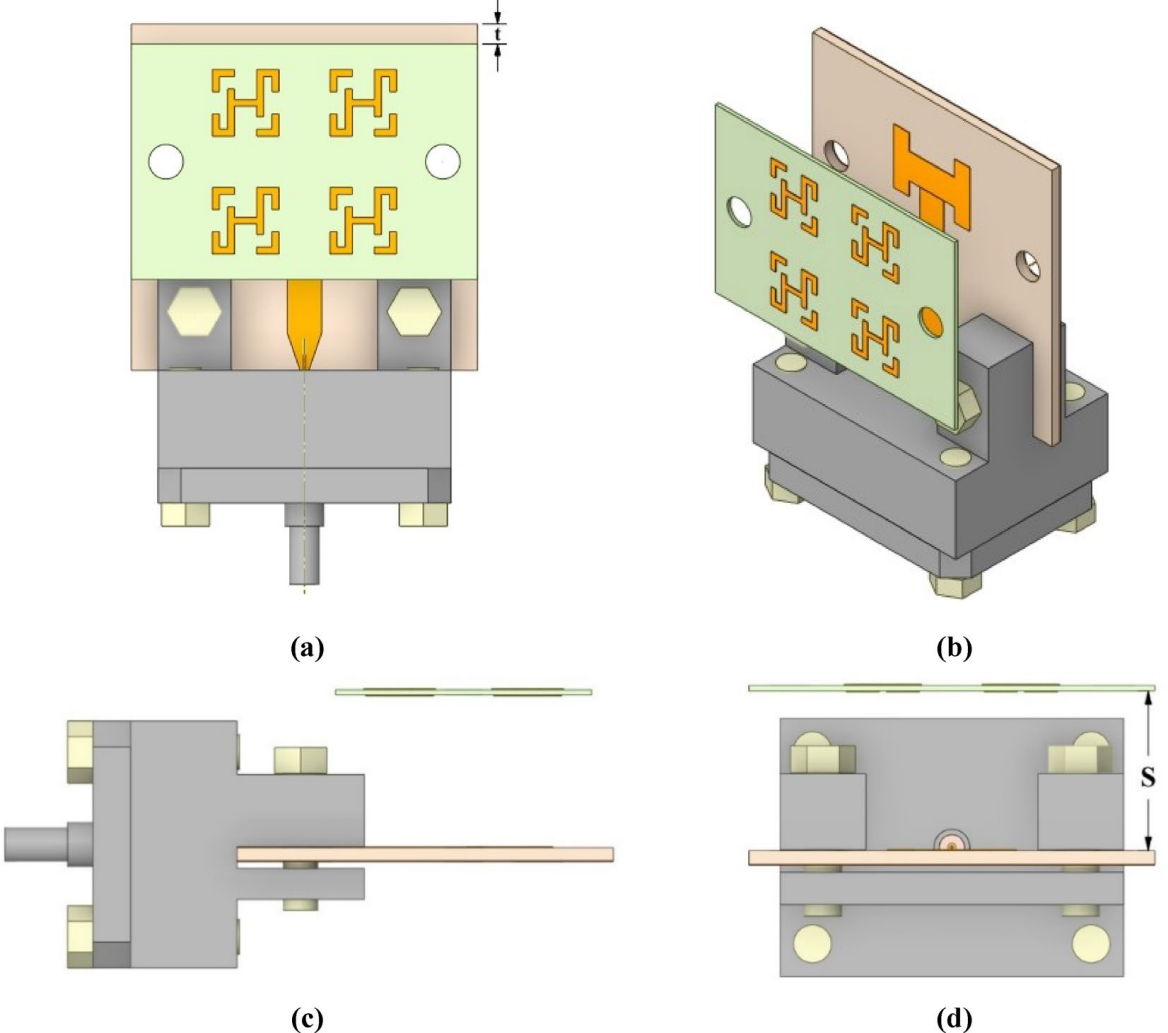
Layout of the proposed metamaterial-based antenna: (**a**) top view, (**b**) 3D layout, (**c**) side perspective, and (**d**) front view.

**Fig. 8:**
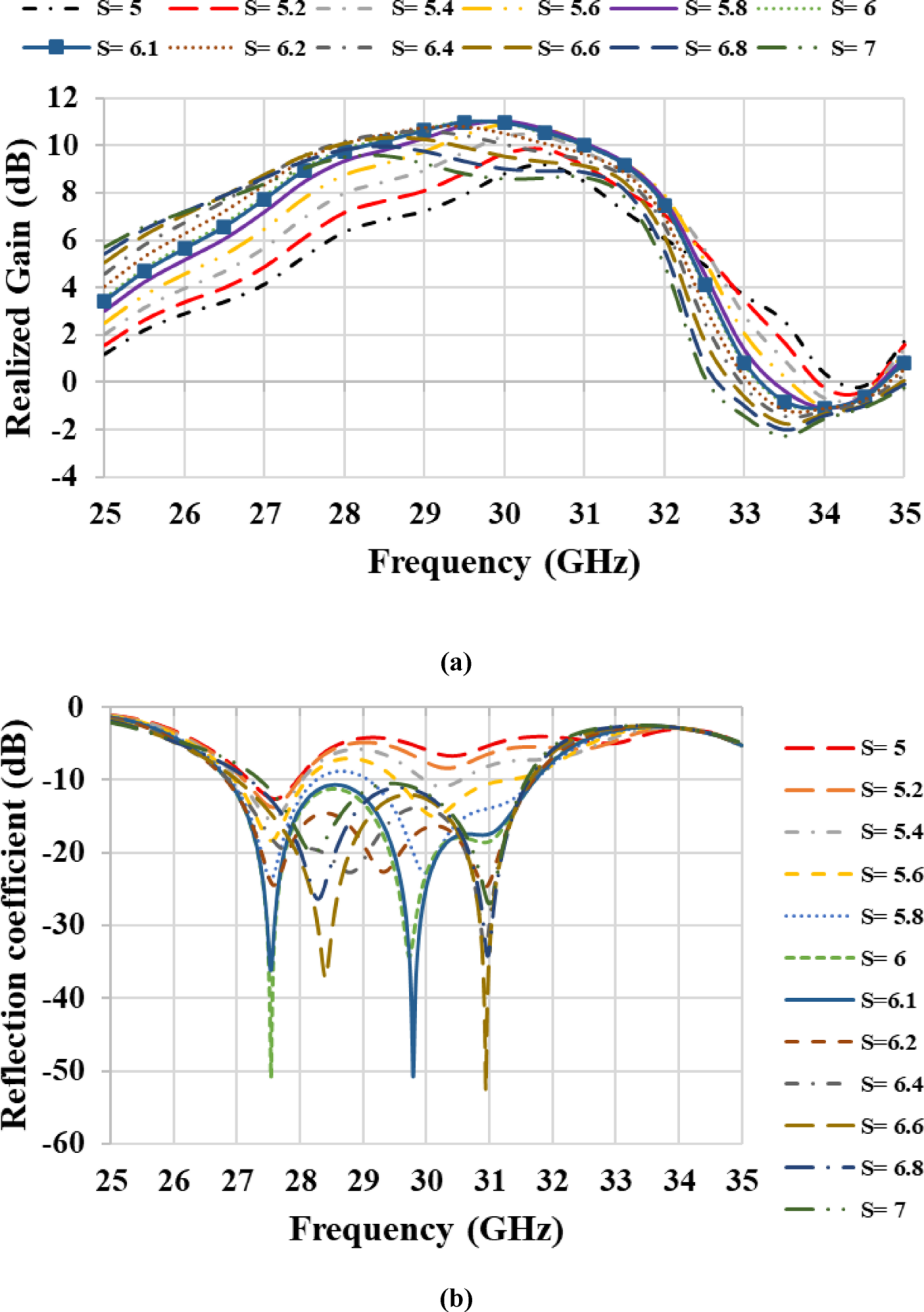

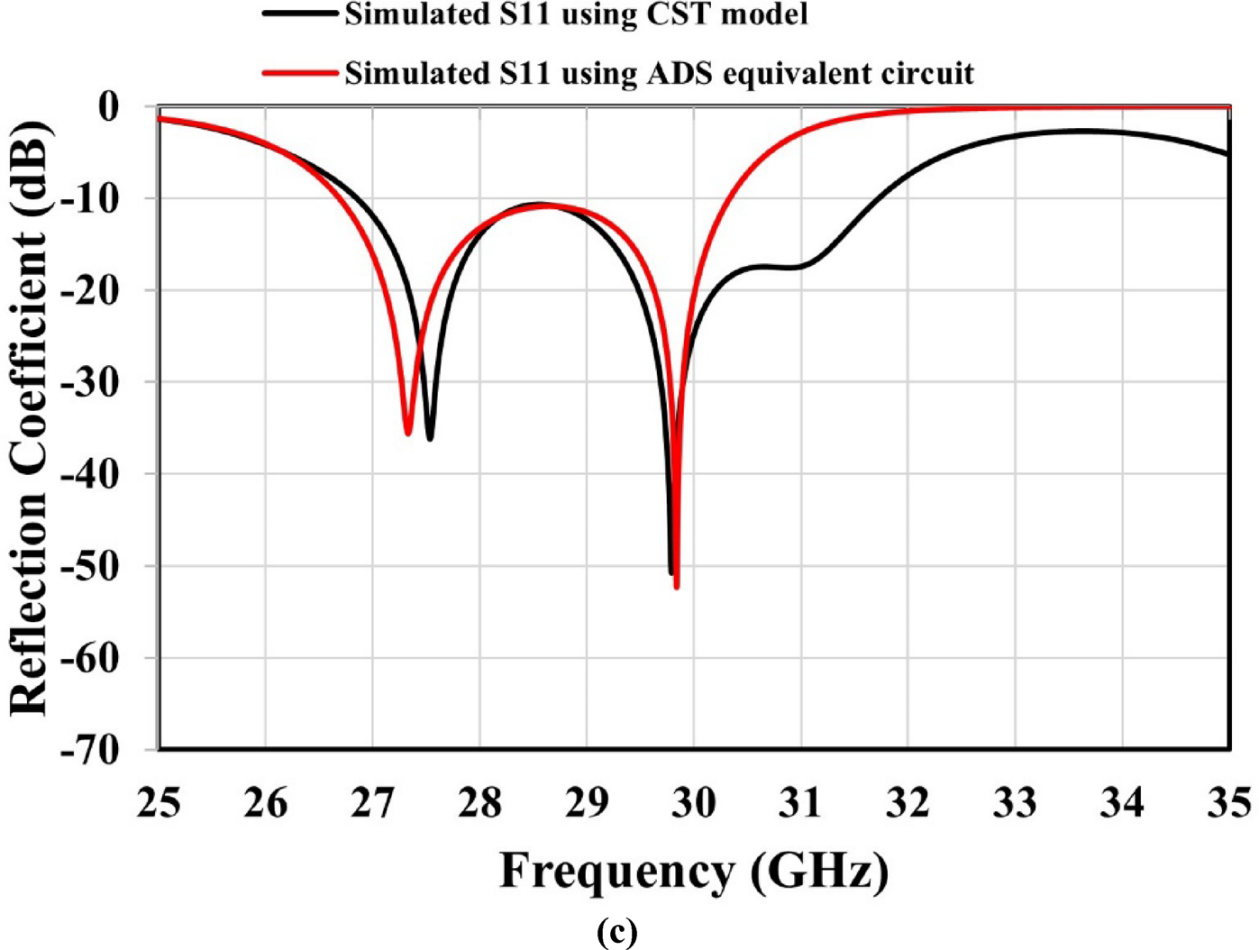
Simulated performance of the proposed metamaterial-based antenna: (**a**) realized gain versus frequency at different air-gap heights, (**b**) reflection coefficient versus frequency at different air-gap heights, and (**c**) reflection coefficient versus frequency at an air-gap height of 6.1 mm.

**Fig. 9:**
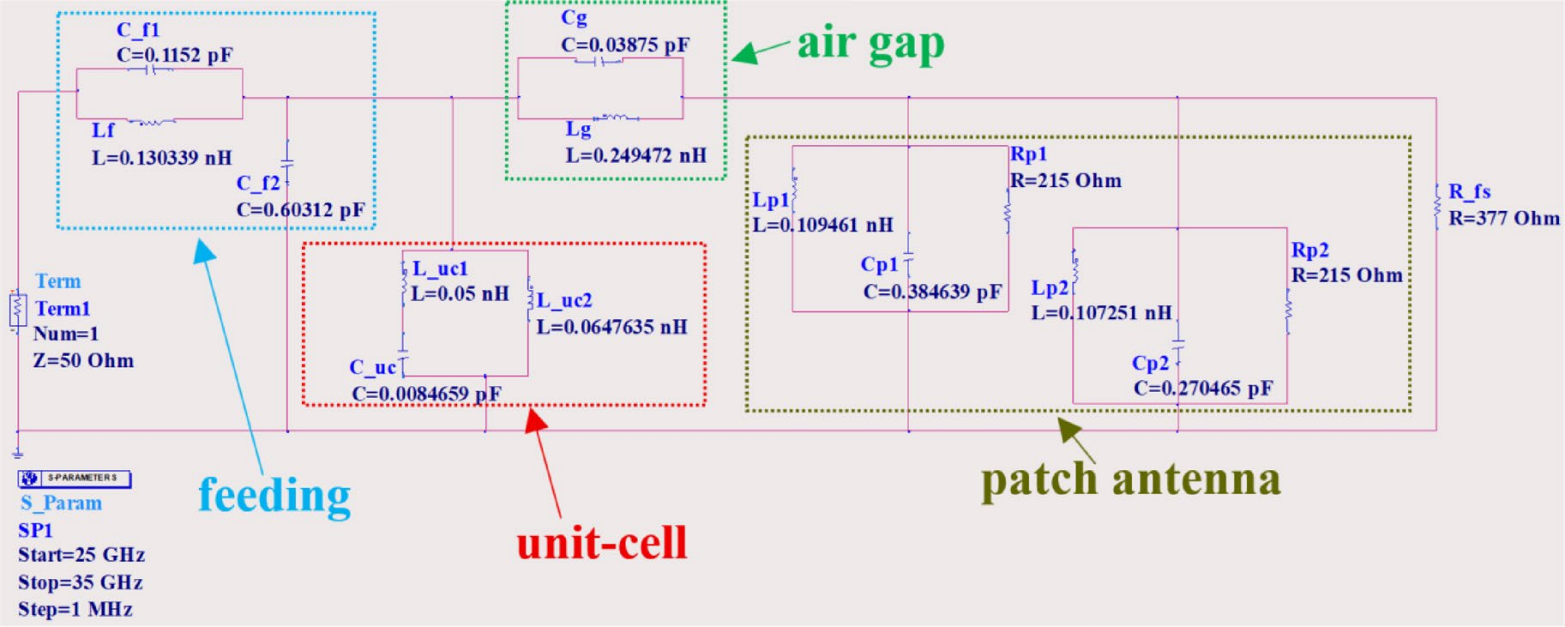
Equivalent circuit model in ADS for the proposed MTM-based antenna.

The metamaterial-based antenna configuration consists of an MTM layer positioned above a microstrip patch antenna and separated by an air gap, as shown in Fig. 7. A single MTM layer composed of a 2 × 2 unit cell array was designed, with overall dimensions of 10.2 mm × 15 mm. The selected periodicity of 5.1 mm (< λ/2 over 26.85–31.72 GHz) suppresses grating lobes and maintains effective medium behavior. Larger spacing introduces grating lobes, while small spacing leads to strong mutual coupling and distortion of the unit-cell response. Therefore, the chosen periodicity provides an appropriate balance between coupling and effective medium behavior^30^. The separation distance **(S)** between the antenna and the MTM layer was optimized to be 6.1 mm to achieve maximum gain and the widest targeted frequency band, as illustrated in Fig. 8(a) and 8(b). The simulated reflection coefficient of the metamaterial-based antenna is depicted in Fig. 8(c), illustrating its frequency response. The plot clearly indicates multiple resonant frequencies where the reflection coefficient drops significantly, indicating good impedance matching and efficient power transfer at these points. The proposed antenna achieves reflection coefficients below − 10 dB over the frequency band of 26.85–31.72 GHz, corresponding to a bandwidth of approximately 4.9 GHz, with reflection coefficients dropping below − 36 dB and − 50 dB within this frequency band, confirming the design’s excellent impedance matching characteristics. These results ensure that the proposed design is well-suited for wideband applications. Furthermore, the proposed metamaterial-based antenna was modeled in ADS using an equivalent circuit, as shown in Fig. 9. The launcher connector and microstrip feed line are represented by a parallel LC resonant circuit, which captures their combined inductive *Lf* and capacitive *C_f1* parasitic effects at the interface. The output of this parallel resonant circuit is connected to ground through a shunt capacitance *C_f2*, which models the fringing fields and capacitive coupling to the ground plane. Due to the complex structure of the launcher connector, the values of *Lf*, *C_f1*, and *C_f2* were obtained through tuning and optimization tools in ADS. The patch antenna exhibits two dominant resonances at 27.8 GHz and 30.7 GHz, as illustrated in Fig. 2. Each resonance is modeled as a parallel RLC branch, representing a radiating mode of the patch antenna. The resonant frequency ( $\:{F}_{R}$ ) is obtained from the minima of the simulated |S_11_|, and the bandwidth ($\:BW$) of each resonance is determined from the |S_11_| response by identifying the two frequencies *f*_*1*_ and *f*_*2*_ around the resonant frequency $\:{F}_{R}$ at which |S_11_| = −10 dB. The bandwidth is then defined as $\:BW={f}_{2}-{f}_{1}$. The quality factor is calculated as $\:Q={F}_{R}/BW$. The inductance, radiation resistance, and capacitance of the patch antenna are obtained using^31^:

19 $$L=\frac{{imag\left( {Z{}_{{11}}} \right)}}{{2\pi {F_R}}},$$

20 $$R=2\pi {F_R}QL,$$

21 $$C=\frac{1}{{{{\left( {2\pi {F_R}} \right)}^2}L}}.$$

Where the real and imaginary parts of the input impedance $\:{Z}_{11}$ vary with frequency. The patch antenna is modeled using two resonant branches: the first branch consists of inductance $\:Lp1$, capacitance $\:Cp1$ and the radiation resistance of $\:Rp1$, while the second resonance branch comprises inductance $\:Lp2$, capacitance $\:Cp2$, and radiation resistance $\:Rp2$. The initial values for the inductances, capacitances, and radiation resistances of the two resonant branches are calculated using the relations defined in Eqs. (19), (20), and (21).

The MTM layer behaves as a passive reactive superstrate that reshapes the current distribution and improves radiation characteristics such as bandwidth and gain. Since the MTM layer does not directly radiate power into free space like the patch, its equivalent circuit is modeled using only reactive elements (inductance and capacitance), without a radiation resistance term. The top layer of the proposed MTM unit cells is modeled as a series combination of inductances $\:L\_uc1$ and capacitance $\:C\_uc$, while the bottom layer is represented by inductance $\:L\_uc2$. The microstrip inductances $\:L\_uc1$ and $\:L\_uc2$ are initially estimated using the following formula^32^:

22 $$L\_us = \frac{{60l}}{C}ln\left\lceil {\frac{{8s}}{w} + \frac{w}{{4s}}} \right\rceil .$$

Where $\:l$ and $\:w$ denote the length and width of the microstrip line, respectively. Additionally, the coplanar capacitance generated by the gaps between the conductors on the top layer of the unit cell is initially calculated using^33^:

23 $$C\_us = \frac{{\varepsilon _{r} l{ln}\left[ { - \frac{2}{{\sqrt[4]{{1 - g^{2} /\left( {g + 2w} \right)^{2} - 1}}}}\left( {\sqrt[4]{{1 - g^{2} /\left( {g + 2w} \right)^{2} + 1}}} \right)} \right]}}{{377\pi C}}$$

Where $\:g$ is the gap between the flat conductor. The air gap between the patch and the MTM layer is realized by an inductance $\:Lg$ and a capacitance $\:Cg$, which represent the magnetic and electric energy stored in the gap region, respectively. The capacitance $\:Cg$ accounts for the electric field coupling and is initially estimated using the parallel-plate approximation:

24 $$Cg = \frac{{\varepsilon _{0} A}}{S}$$

Here, $\:{\epsilon\:}_{0}$ represents the free-space permittivity. The capacitance is primarily determined by the geometrical overlap area between the patch and the MTM layer (A) and separation distance (S). The inductance $\:Lg$ depends on the magnetic field, which extends beyond the physical region and is highly sensitive to the current distribution. Consequently, analytical expressions cannot accurately estimate the air-gap inductance $\:Lg$ and it is determined through optimization in ADS. Finally, the impedance $\:R\_fs=377{\hspace{0.17em}}{\Omega\:}$ is included in parallel with the overall circuit to account for radiation into free space. The complete equivalent circuit was implemented and optimized in ADS, as illustrated in Fig. 9. The component values were fine-tuned to ensure that the simulated S-parameters closely correspond to those obtained from CST full-wave simulation, as shown in Fig. 8(c).

**Fig. 10:**
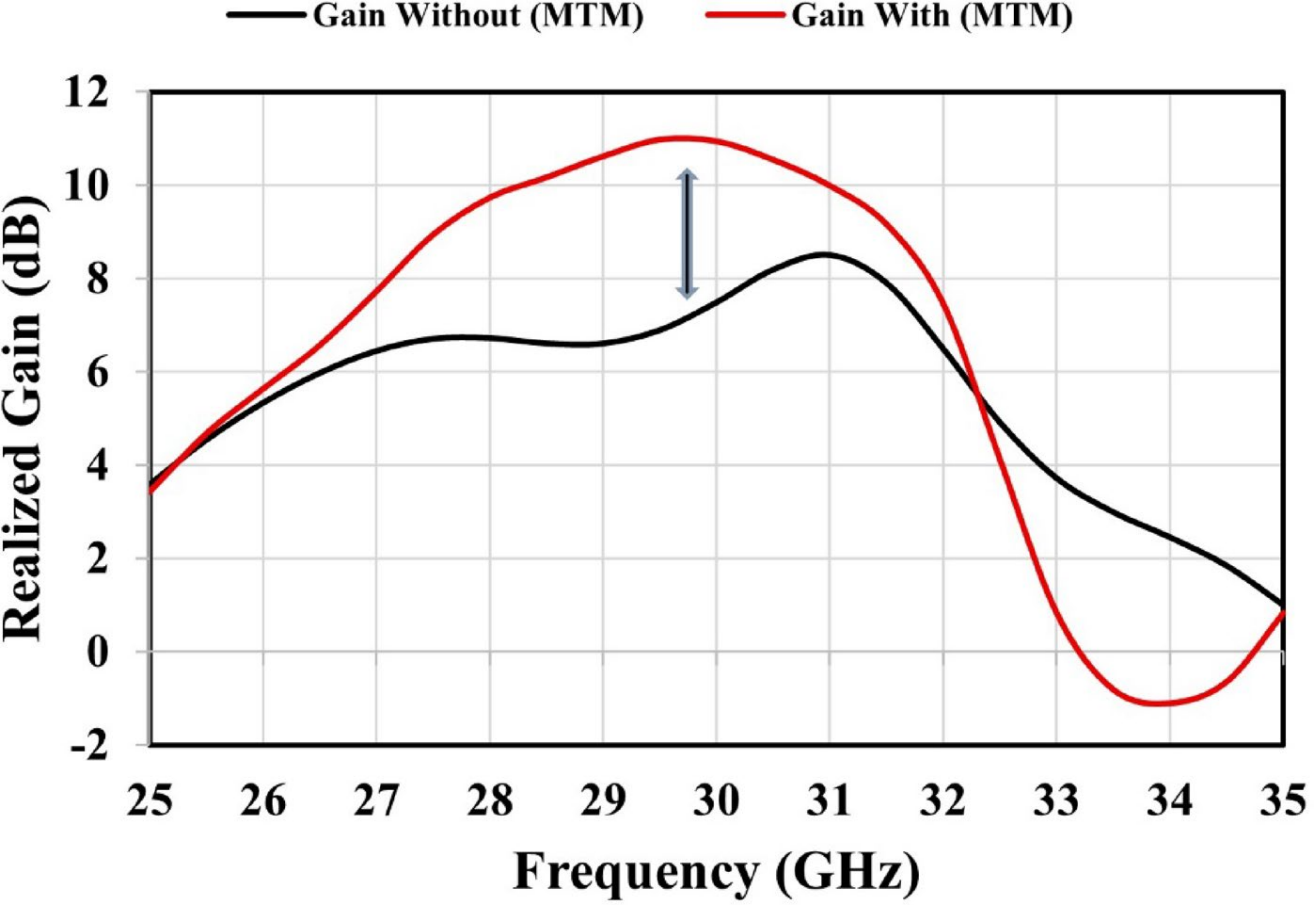
Realized gain of the proposed antenna without and with MTM.

**Fig. 11:**
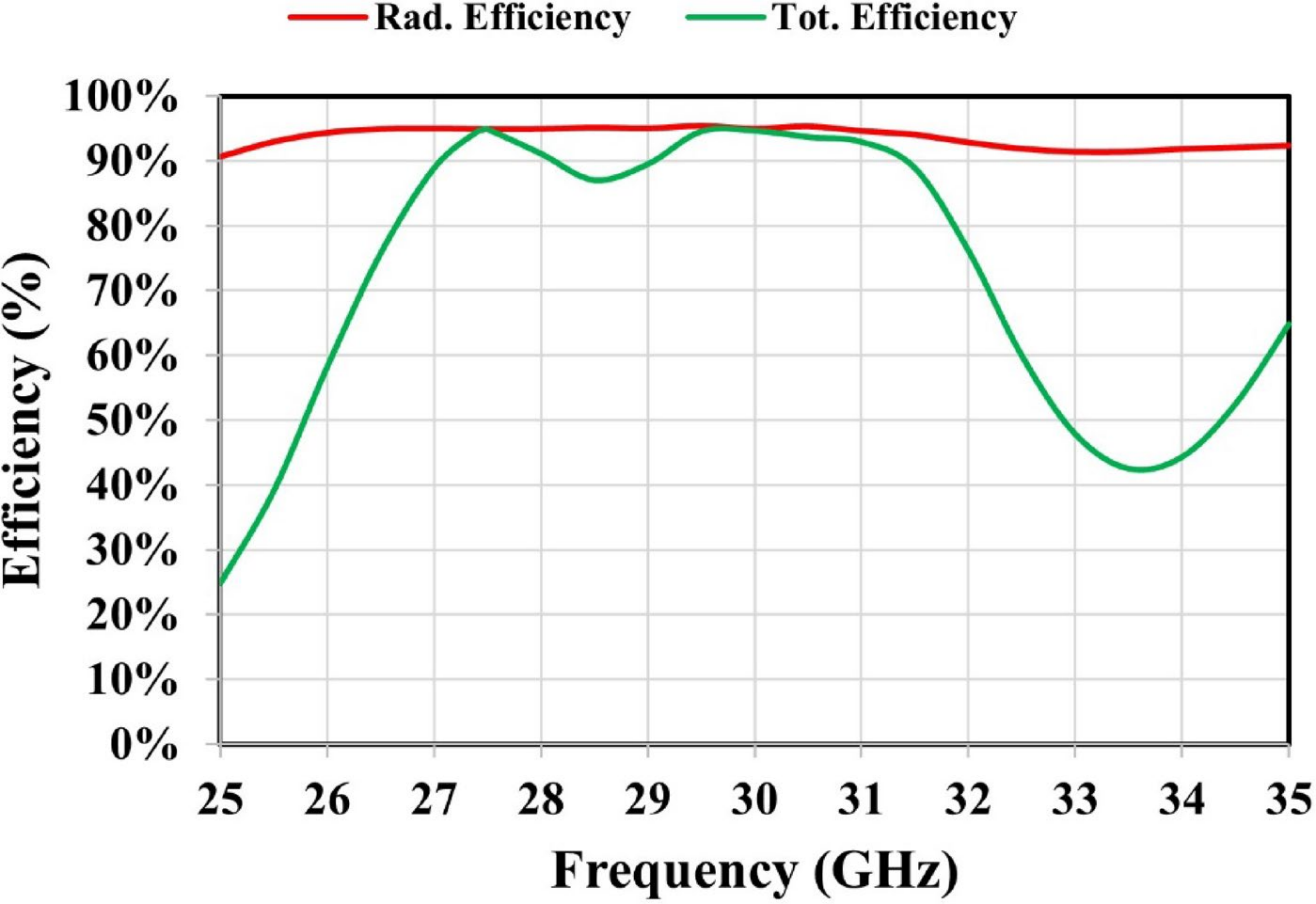
Efficiency of the metamaterial-based antenna.

Figure 10 illustrates that the maximum gain of the MTM 2 × 2 single-layer unit cell array reaches 11 dBi, representing an improvement of approximately 2.5 dB over the 8.5 dBi achieved by the antenna without the MTM layer. In the subsequent section, the proposed antenna with an integrated single layer of the MTM 2 × 2 unit cell array is experimentally validated. Figure 11 presents the simulated radiation and total efficiencies of the proposed metamaterial-based antenna. At 27.4 GHz, the proposed antenna achieves radiation and total efficiencies of approximately 94.8% and 94.4%, respectively, while at 30 GHz, these efficiencies increase to about 95% and 94.5%, respectively.

**Fig. 12:**
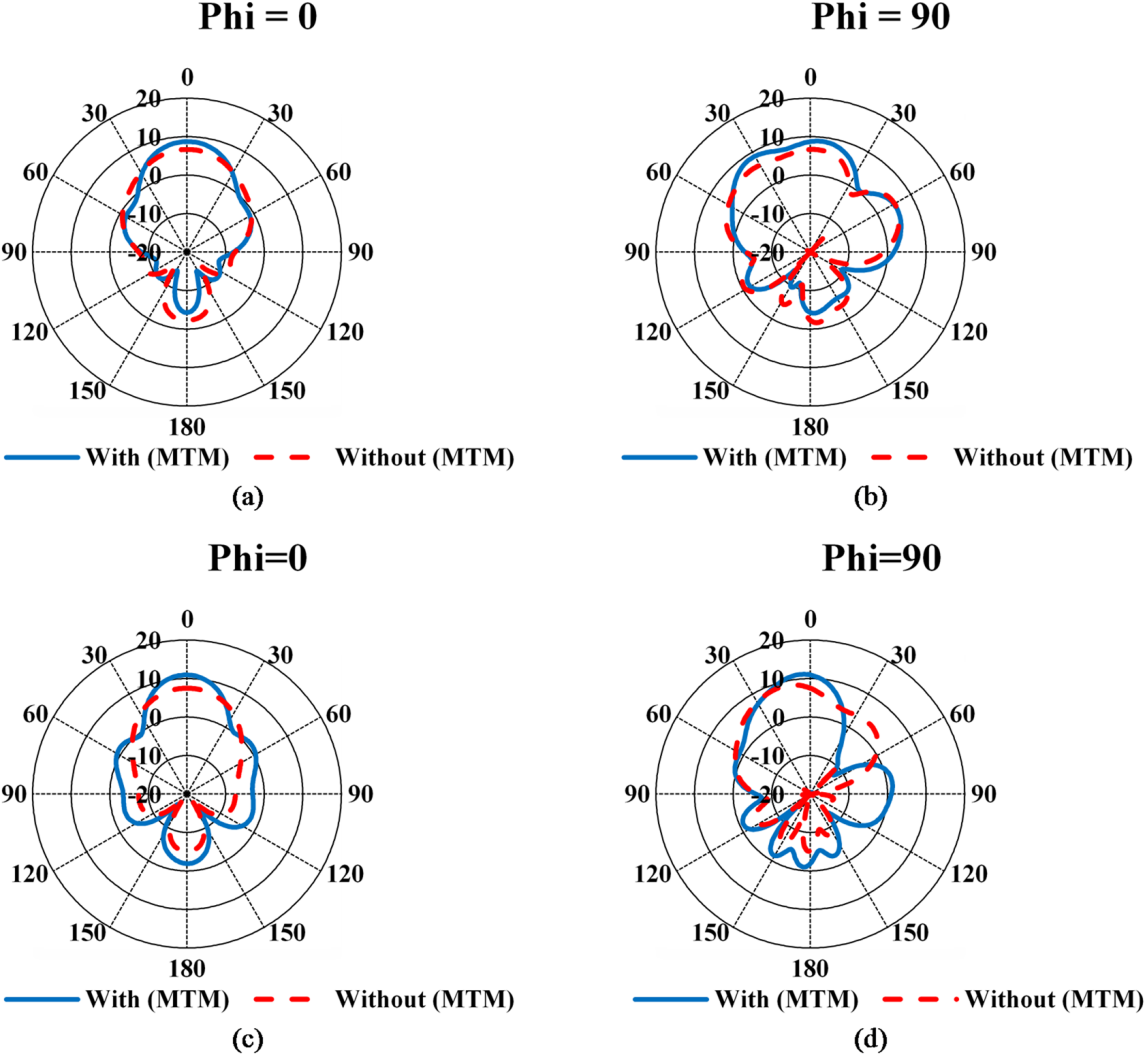
Simulated XZ- and YZ-planes radiation patterns of the proposed antenna without and with MTM: (**a**) XZ-plane at 27.4 GHz, (**b**) YZ-plane at 27.4 GHz, (**c**) XZ-plane at 30 GHz, and (**d**) YZ-plane at 30 GHz.

Figure 12 presents the simulated radiation patterns of the antenna without and with the incorporation of MTM at 27.4 GHz and 30 GHz, evaluated at **Phi** = 0° and 90°, to demonstrate the impact of MTM on antenna performance. The simulation results clearly indicate that the incorporation of MTM enhances the radiation characteristics and increases the antenna gain. Furthermore, the sidelobe levels of the metamaterial-based antenna remain below − 10 dB within these frequency bands. The simulated antenna efficiency was also analyzed at these frequencies, and the corresponding performance parameters with and without MTM integration are summarized in Table 3.

**Table 3:** Simulated performance parameters of the proposed antenna with and without MTM.

	Frequency (GHz)		Realized Gain (dBi)	Dir. (dBi)	Rad. Efficiency	Tot. Efficiency
Ant. without (MTM)	27.85		6.7	7.09	95%	94%
	30.72		8.1	9.73		
Ant. with (MTM)	Bandwidth From 26.85 to 31.72	27.4	9	9.19	94.8%	94.4%
		30	11	11.4	95%	94.5%

### Characteristic mode analysis of the proposed metamaterial-based antenna

**Fig. 13:**
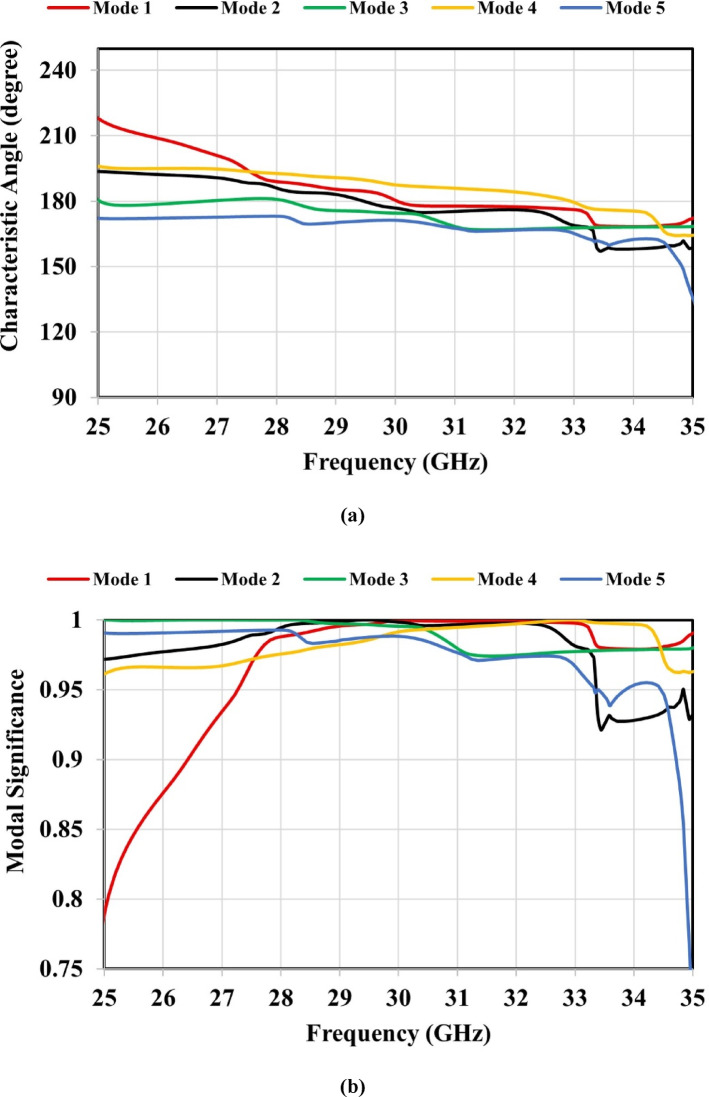

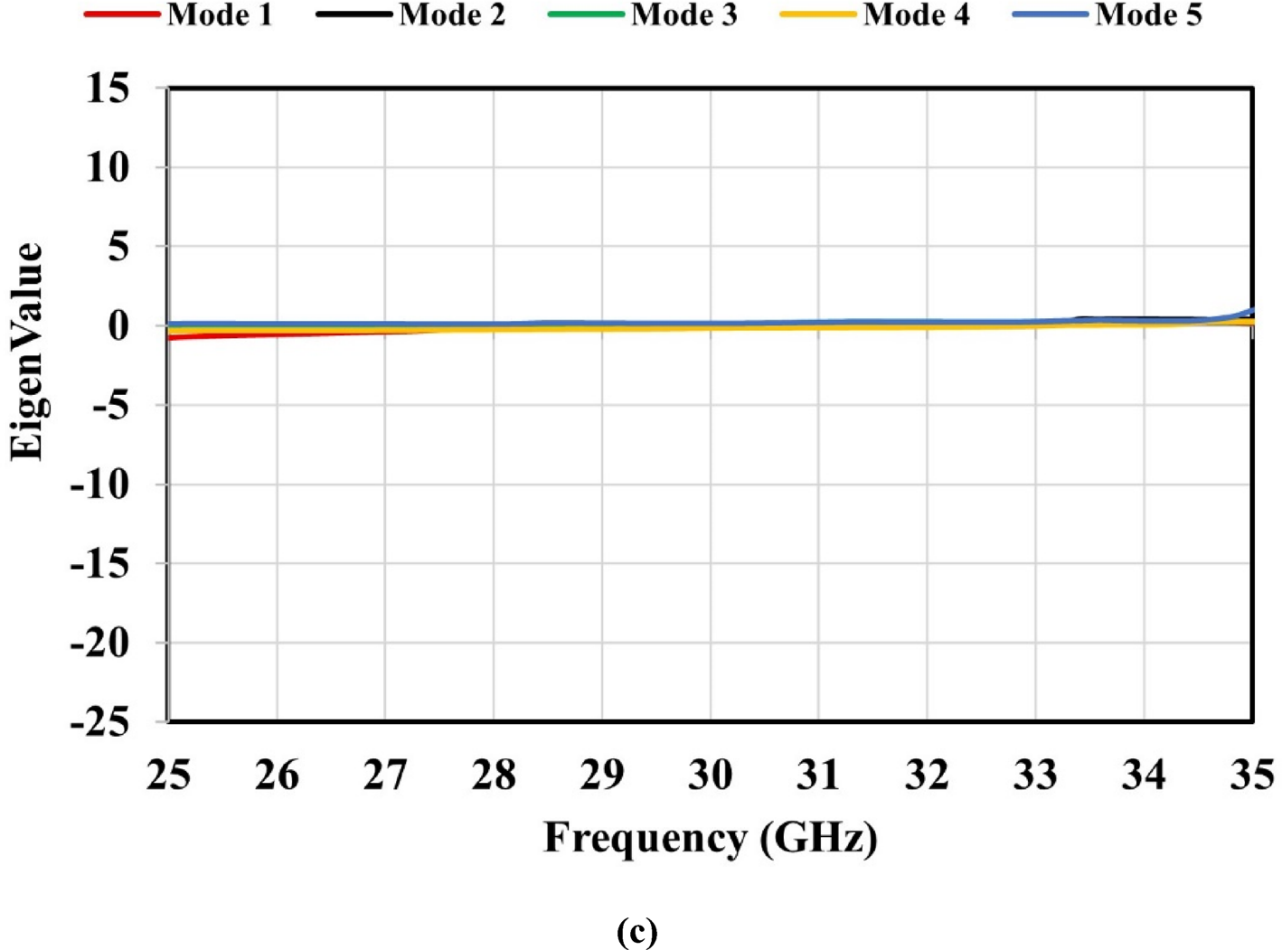
Characteristic mode analysis of the metamaterial-based antenna: (**a**) characteristic angle, (**b**) modal significance, and (**c**) eigenvalue.

This section investigates the resonance characteristics of the proposed metamaterial-based antenna without applying external excitation, using CMA parameters such as the characteristic angle, modal significance, and eigenvalue. These parameters were obtained through the CMA tool available in the CST Integral Equation Solver. A total of five characteristic modes were analyzed for the proposed metamaterial-based antenna. In CMA, each mode corresponds to a distinct current distribution (eigenvector) on the structure. The eigenvalue ($\:{\lambda\:}_{n}$) of a mode varies with frequency, and resonance occurs when $\:{\lambda\:}_{n}$ crosses zero; at this condition, the mode radiates most efficiently because the stored reactive energy becomes zero. When the magnitude of the eigenvalue $\:\mid\:{\lambda\:}_{n}\mid\:$ is close to zero, the mode is near resonance and radiates efficiently, whereas large $\:\mid\:{\lambda\:}_{n}\mid\:$ values indicate that the mode is far from resonance and contributes weakly to radiation^34^. Figure 13(a) shows that Mode 1 crosses 180^◦^ and resonates at 30 GHz, while Mode 2 resonates at 29.5 GHz. Additionally, Modes 3 and 4 resonate at 28 and 33 GHz, respectively, but no resonances are observed for Mode 5. Figure 13(b) illustrates that all five modes exhibit modal significance values exceeding 0.707 within the 25–35 GHz frequency band. As shown in Fig. 13(c), all five modes have $\:\mid\:{\lambda\:}_{n}\mid\:$ values approaching zero across the frequency band; consequentially, the modal significance values **(**$\:\boldsymbol{M}\boldsymbol{S}\:=\:\frac{1}{\left|1+\boldsymbol{j}{\boldsymbol{\lambda\:}}_{\boldsymbol{n}}\right|}$**)** remain above 0.707. This confirms that all modes, including Mode 5, contribute to the antenna’s radiation.

**Fig. 14:**
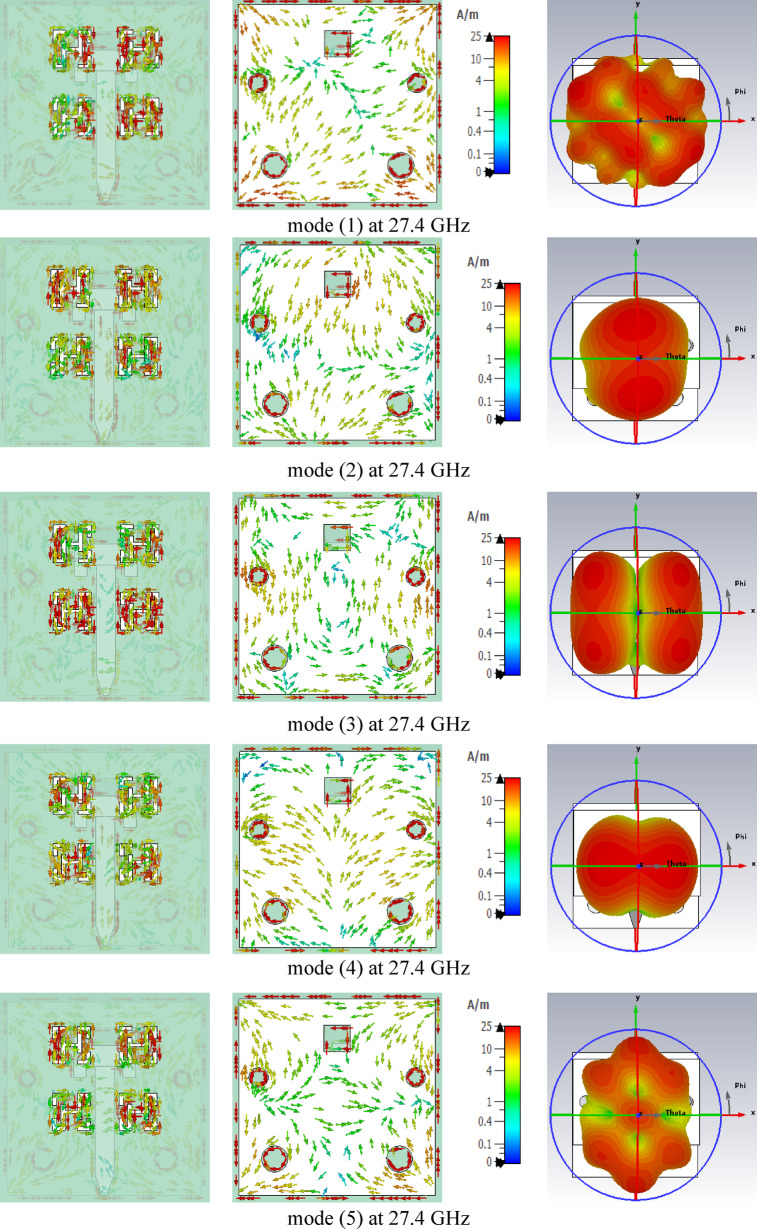
Simulated current distributions and related directivity patterns of the five modes in the proposed metamaterial-based antenna at 27.4 GHz.

**Fig. 15:**
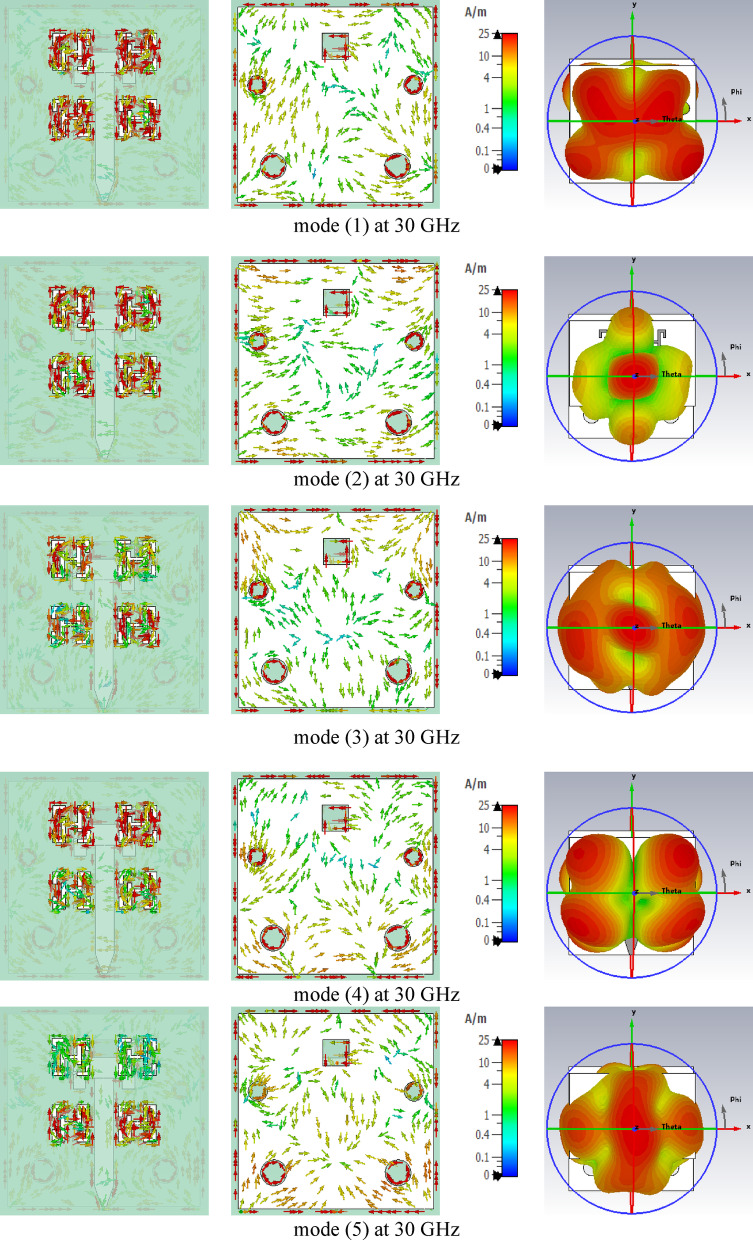
Simulated current distributions and corresponding directivity patterns of the five characteristic modes in the proposed metamaterial-based antenna at 30 GHz.

The surface current distributions of the five modes were examined at the resonance frequencies of 27.4 GHz and 30 GHz, as illustrated in Figs. 14 and 15. At 27.4 GHz, Modes 1, 2, 3, and 4 exhibit capacitive behavior, whereas Mode 5 is inductive. Moreover, all modes contribute to the total radiation pattern at 27.4 GHz; however, the radiation patterns of Modes 1 and 5 display more lobes due to greater variations in their surface current distributions compared to Modes 2, 3, and 4. At 30 GHz, Mode 1 demonstrates magnetic resonance with a vertical current distribution, while Modes 2, 3, and 5 are inductive, and Mode 4 is capacitive. All five modes contribute to the overall radiation pattern at 30 GHz. Additionally, as the frequency increases, variations in the surface current distributions become more noticeable, resulting in radiation patterns with multiple lobes for all five modes, as illustrated in Fig. 15. CMA was employed to provide a deeper physical understanding of the proposed antenna, independent of the feeding structure. Specifically, CMA revealed the existence of five natural resonant modes supported by the proposed MTM-based antenna. This modal decomposition enabled clear identification of the contributing modes and explained the bandwidth enhancement mechanism in terms of the coexistence and spectral overlap of these five dominant modes. Furthermore, CMA demonstrated that these modes were simultaneously excited at the resonant frequencies of 27.4 GHz and 30 GHz. The corresponding current distributions confirmed that each mode contributed effectively to radiation, thereby improving the overall gain of the proposed antenna. Therefore, CMA provided essential physical insight into the antenna operation, particularly in terms of mode identification, bandwidth enhancement, and radiation performance, which could not be clearly obtained or interpreted using conventional full-wave analysis alone.

### Fabrication and experimental validation

**Fig. 16:**
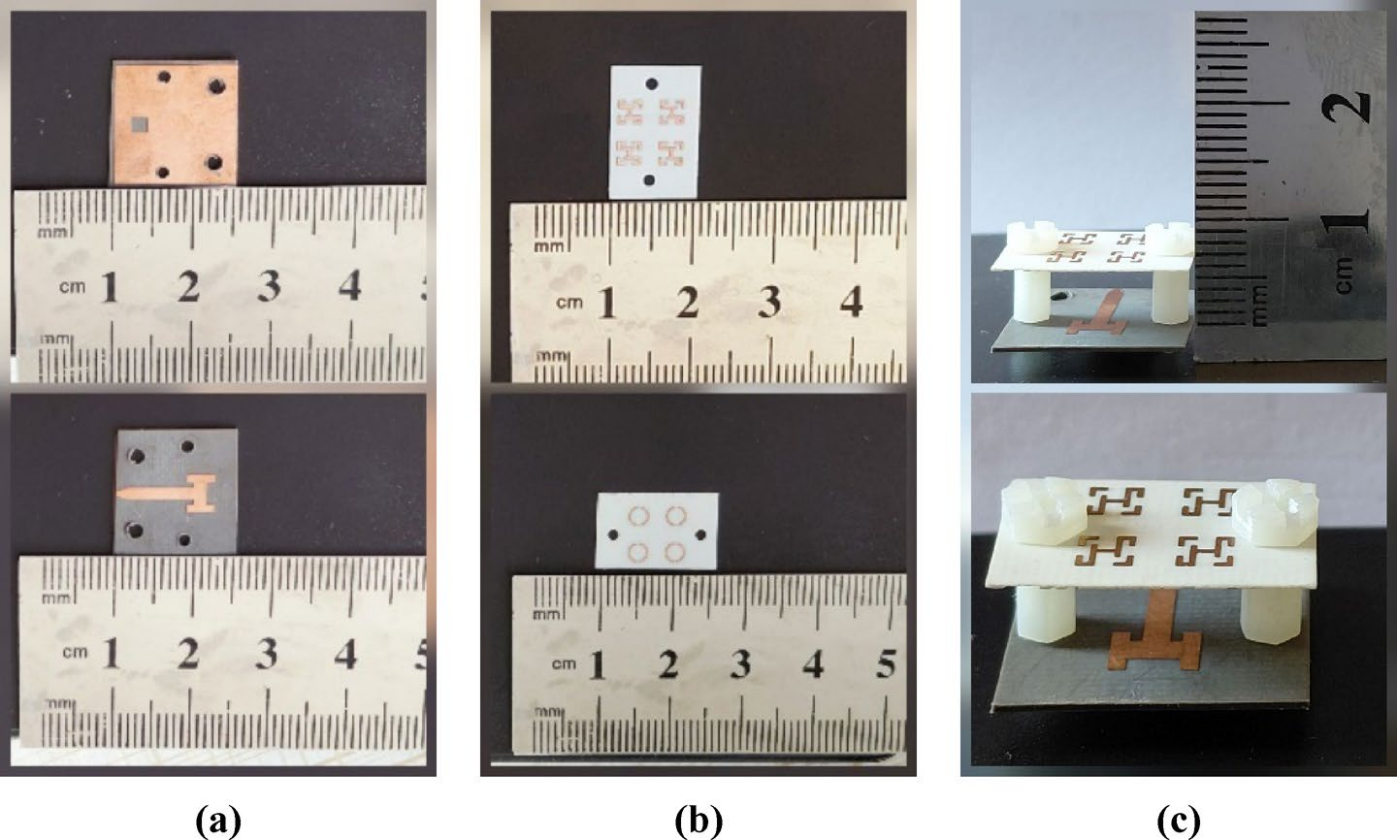
Fabricated MTM-based antenna prototype, including (**a**) patch top and bottom views, (**b**) MTM layer top and bottom views, and (**c**) a three-dimensional view of the complete antenna assembly.

**Fig. 17:**
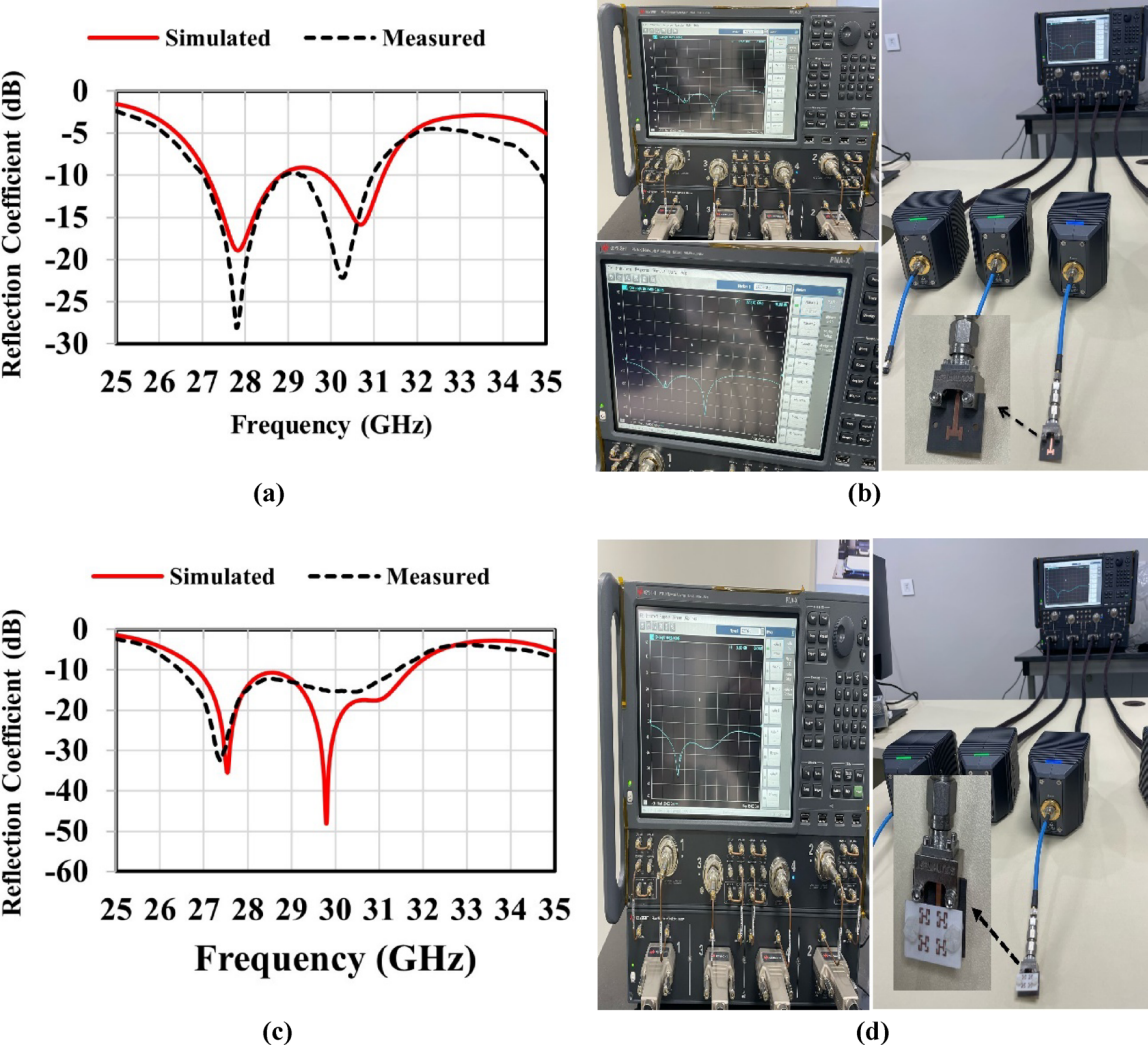
Reflection coefficient results (simulated and measured) of the proposed antenna: without the MTM layer (**a**,** b**) and with the MTM layer (**c**,** d**).

**Fig. 18:**
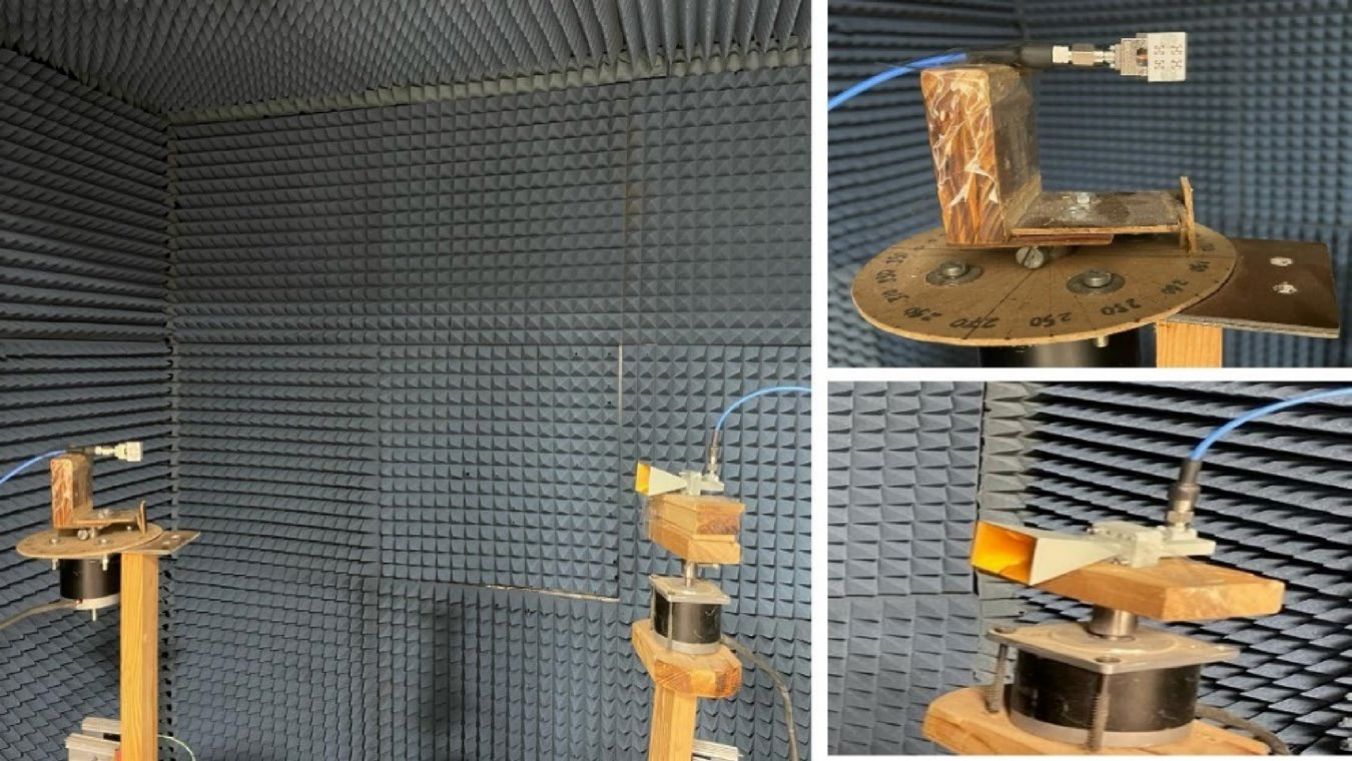
Measurement setup for evaluating the radiation pattern and gain of the metamaterial-based antenna.

**Fig. 19:**
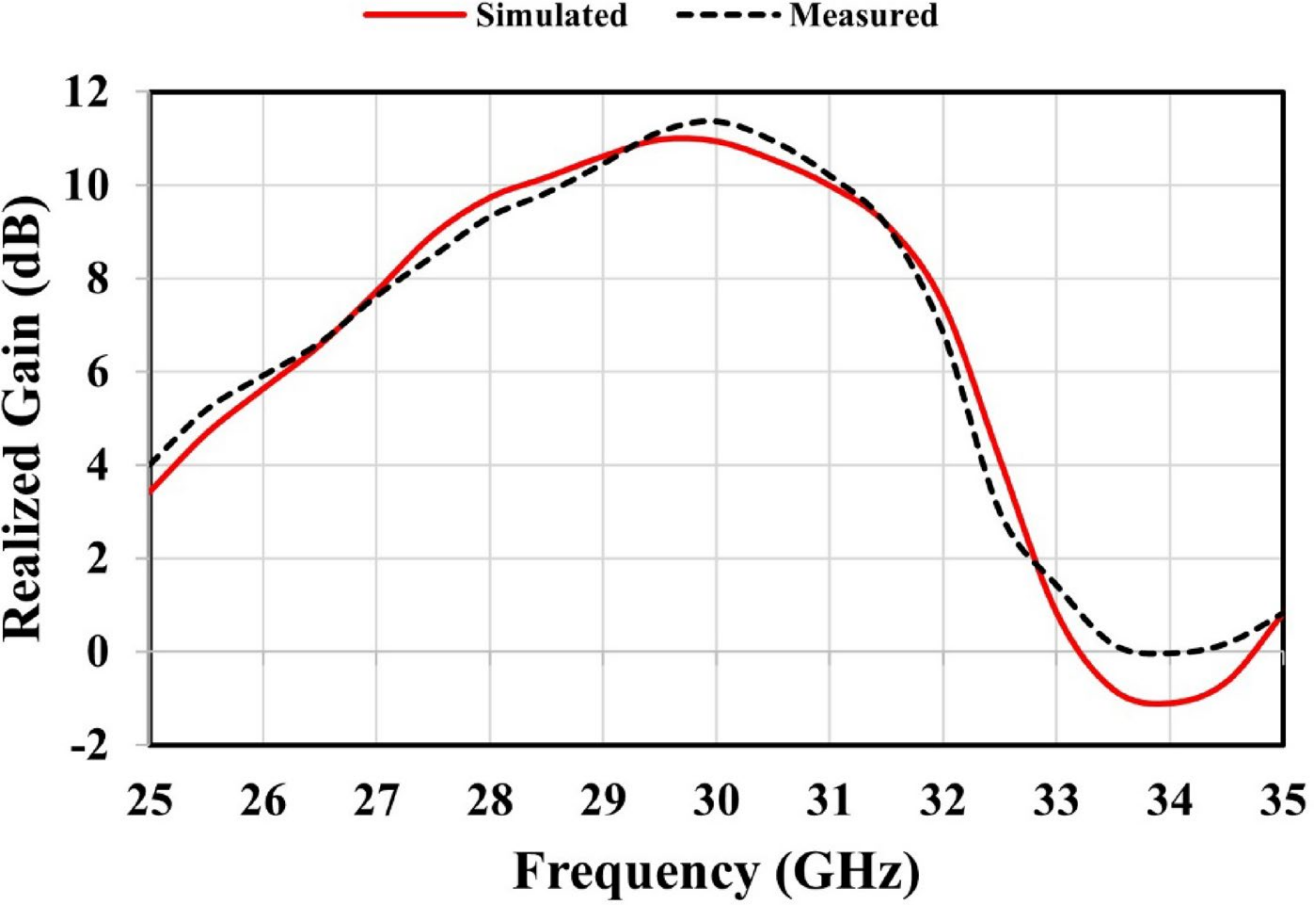
Realized gain of the metamaterial-based antenna, showing both simulated and measured results.

**Fig. 20:**
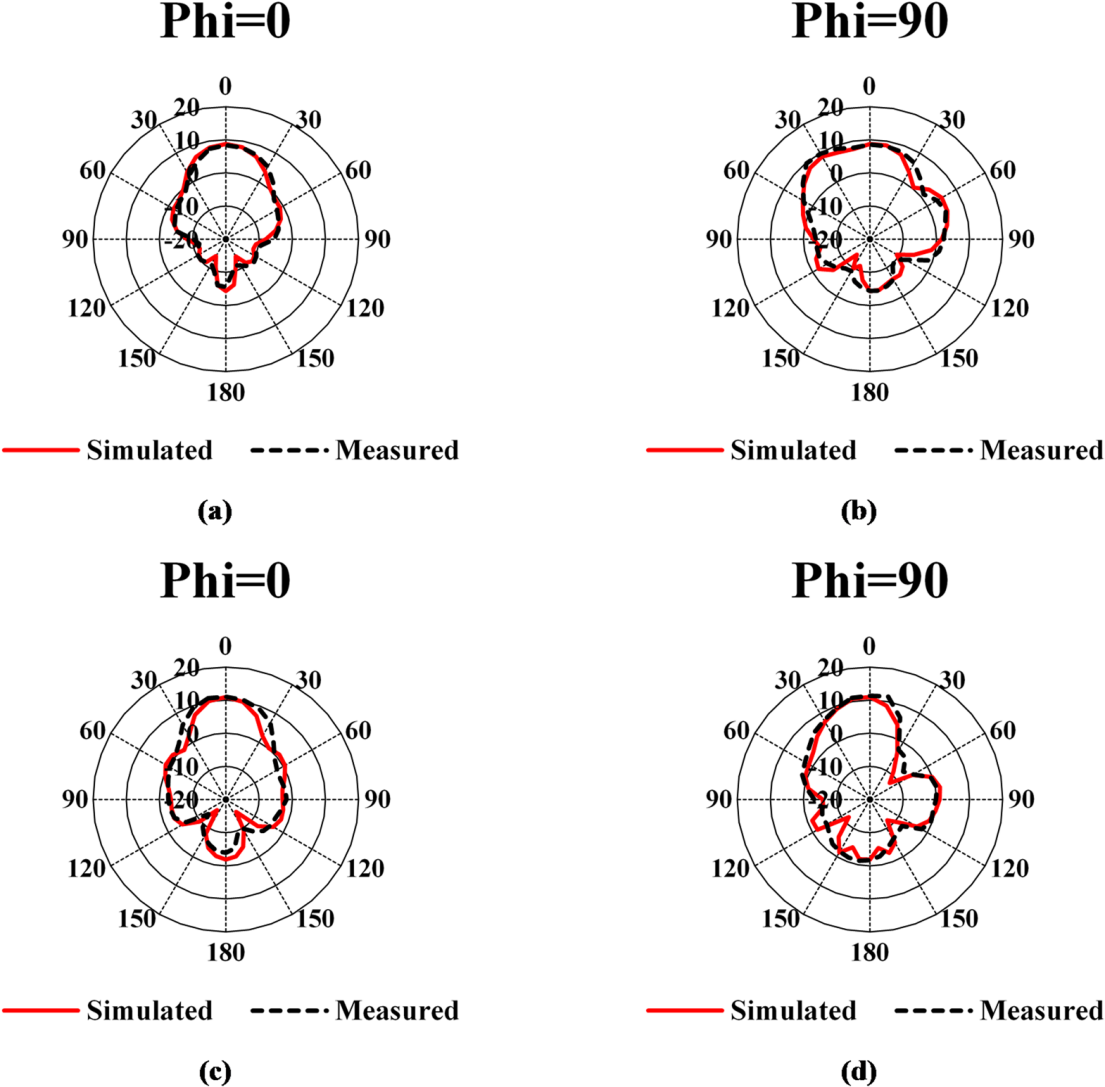
Simulated and measured XZ- and YZ-planes radiation patterns of the antenna integrated with MTM: XZ-plane at 27.4 GHz (**a**), YZ-plane at 27.4 GHz (**b**), XZ-plane at 30 GHz (**c**), and YZ-plane at 30 GHz (**d**).

The MTM-based antenna, which consists of a 2 × 2 array of metamaterial unit cells, was fabricated and subsequently measured. The proposed unit cell array and the patch antenna were designed using Rogers’s substrates RO4003C and RO5880, respectively. Figure 16 presents photographs of the proposed antenna prototype, taken both before and after assembly completion. A 6.1 mm air gap separates the antenna and unit cell substrates, maintained by precisely designed 3D-printed spacers, as shown in Fig. 16(c). Reflection coefficient measurements for both the patch antenna without and with the MTM layer were performed using a PNA-X N5244B vector network analyzer (VNA). The simulated and measured results indicate that the patch antenna without the MTM layer exhibits dual resonances centered at 27.8 GHz and 30.7 GHz, demonstrating good impedance matching, as illustrated in Figs. 17(a) and (b). The experimental and simulated results for the MTM-based antenna show strong agreement, with minor discrepancies attributed to the compact antenna structure and the feed line–connector interface. The proposed MTM-based antenna achieves wideband performance with impedance matching better than − 40 dB. The measurements reveal a bandwidth exceeding 4.5 GHz, spanning from 26.85 GHz to 31.72 GHz, with peak performance observed at approximately 27.4 GHz and 30 GHz as shown in Fig. 17 (c) and (d). Figure 18 shows the setup in the anechoic chamber, where the gain and radiation pattern were measured using a standard LB-180-400 gain horn antenna. The realized gain of the MTM-based antenna prototype was evaluated across the operating frequency range, as presented in Fig. 19. The results indicate that the MTM-based antenna achieved a maximum gain of 11.4 dBi within the operational frequency range. Simulated and measured XZ and YZ planes radiation patterns at 27.4 GHz and 30 GHz are shown in Fig. 20. Strong agreement is observed between simulations and measurements, with minor deviations attributed to fabrication tolerances and slight reflections from the launcher connector.

**Table 4:** Performance comparison between the proposed design and previously reported designs operating in the millimeter-wave frequency range.

Ref.		Frequency (GHz)	B.W (GHz)	Max. Realized Gain (dBi)	Thickness / ε_*r*_	Size ($\:{\boldsymbol{m}\boldsymbol{m}}^{3}$)	Radiation efficiency (%)
This Work		27.4	(26.85–31.72) 4.81	11.4	Element 0.508 /2.2	15 × 15 × 0.508	95
		30			MTM 0.2 / 3.38	10.2 × 15 × 0.2	
^9^	Ant.	28	3.8	10.3	0.203 / 3.55	12 × 12 × 0.203	90
	FSS	28	5.3		0.5 / 2.2	25 × 25 × 5	
^21^		26.4	3.8	10	Ant. 0.508 / 2.2	20 × 21 × 0.508	91.2
		27.6	2.9		MS. 1.575 / 2.2	11 × 21 × 1.575	
^35^		23.9–30.7	6.8	9.4	0.51 / 2	19.76 × 19.76 × 0.51	87
^36^		26.62–29.79	3.17	13	0.508 /2.2	18 × 14.5 × 0.508	94
^37^		28.6	(26.2–30) 3.8	11	0.508 /2.2	NA	NA
^38^		41	(23.1–44.8) 21	11.21	0.8 / 3.55	32 × 10 × 0.8	64
^39^		28 and 38	(24–28.8) 4.8	7.8 and 6	0.787 / 2.2	18 × 9.2 × 0.787	NA
			**(**36.6–40.8**) 4.2**				
^40^		9.5	(8.76–10.74) 1.98	8.1	1.6 / 4.4	27 × 27 × 1.6	91.2
^41^		28	2.6 (26.45–29.25)	9.45	0.254 / 10.2	30 × 30 × 0254	NA

The results presented in this paper show that the proposed MTM-based antenna is well-suited for advanced wireless communication applications. Table 4 summarizes a comparison with recent state-of-the-art works published in the literature, many of which utilize metamaterial superstrate unit cells to enhance gain at millimeter-wave frequencies. The results indicate that the proposed MTM-based antenna offers an excellent balance between high gain and compact size, providing a significant increase in gain compared to antennas of similar dimensions while maintaining a noticeably smaller footprint than benchmark designs that have comparable gain levels. Although the design in^36^ reports a higher gain, it occupies a larger size and have a lower bandwidth. The proposed design shows the metamaterial properties and achieves a wide bandwidth of approximately 4.8 GHz, enabled by precise modifications to the unit cell that realize a negative refractive index. Although substantial research on negative metamaterials has been performed, further exploration remain essential, particularly for emerging technologies like 5G and beyond. The proposed MTM-based antenna is a promising candidate for 5G small-cell base station applications. This research advances both the understanding and practical deployment of negative-index metamaterials, creating new prospects for revolutionary communication technologies.

## Conclusion

This study presents a novel double-negative metamaterial (DNG) designed to improve the performance of conventional millimeter-wave antennas. The proposed configuration integrates a metamaterial layer as a superstrate above the microstrip patch antenna, thereby achieving improved gain, wider bandwidth, and increased efficiency. The proposed MTM 2 × 2 unit cell array features a compact design, occupying an area of approximately 10.2 × 15 mm², while the MTM-based antenna measures 15 × 15 × 6.1 mm³. The antenna array attains a peak gain of 11.4 dBi and operates over a wide bandwidth of 26.85–31.72 GHz, with a radiation efficiency of approximately 95%. Significant improvements in radiation patterns on both the XZ and YZ planes at the resonant frequencies of 27.4 GHz and 30 GHz are observed due to the inclusion of the MTM superstrate layer. The proposed MTM-based antenna exhibits excellent agreement between measured and simulated results, confirming its potential for deployment in 5G millimeter-wave networks.

## Data Availability

The datasets used and/or analyzed during the current study available from the corresponding author on reasonable request.

## References

[1] Jijo, B. T. et al. A comprehensive survey of 5G mm-wave technology design challenges. *Asian J. Res. Comput. Sci.* **8**, 1–20. 10.9734/ajrcos/2021/v8i130190 (2021).

[2] Nahar, T. & Rawat, S. J. A review of design consideration, challenges and technologies used in 5G antennas. *Wirel. Pers. Commun.* **129**, 1585–1621. 10.1007/s11277-023-10193-x (2023).

[3] Kamal, M. M. et al. A novel hook-shaped antenna operating at 28 GHz for future 5G mmwave applications. *Electronics* **10**, 673. 10.3390/electronics10060673 (2021).

[4] Althuwayb, A. A. MTM- and SIW-inspired bowtie antenna loaded with AMC for 5G mm-wave applications. Int. J. Antennas Propag. 6658819 (2021). (2021). 10.1155/2021/6658819

[5] Emadian, S. R. & Ghobadi, C. Enhanced double negative metamaterial microstrip antenna: bandwidth, gain improvement, and size reduction for radar applications. *IEEE Access.* **12**, 170079–170088. 10.1109/ACCESS.2024.3498453 (2024).

[6] Li, F., Lv, M., Wang, M. & Jia, Y. An in-band low-radar cross section microstrip patch antenna based on a phase control metasurface. *Electronics* **13**, 1718 (2024).

[7] Esmail, B. A., Koziel, S. & Isleifson, D. Metamaterial-based series-fed antenna with a high gain and wideband performance for millimeter-wave spectrum applications. *Electronics* **12**, 4836. 10.3390/electronics12234836 (2023).

[8] Ajewole, B., Kumar, P. & Afullo, T. J. M. A microstrip antenna using I-shaped metamaterial superstrate with enhanced gain for multiband wireless systems. *Micromachines* **14**, 412. 10.3390/mi14020412 (2023). 36838111 10.3390/mi14020412 PMC9959085

[9] Mohamed, H. A., Edries, M., Abdelghany, M. A. & Ibrahim, A. A. Millimeter-wave antenna with gain improvement utilizing reflection FSS for 5G networks. *IEEE Access.* **10**, 73601–73609. 10.1109/ACCESS.2022.3189651 (2022).

[10] Sansa, I., Nasri, A. & Zairi, H. Bandwidth enhancement of an antenna based on metamaterial using characteristic mode analysis for microwave applications. *J. Eng. Sci. Technol.* **18**, 636–652 (2023).

[11] Rahman, M. M., Islam, M. S., Islam, M. T., Al-Bawri, S. S. & Yong, W. H. Metamaterial-based compact antenna with defected ground structure for 5G and beyond. *Comput. Mater. Contin*. **72**, 2383–2399. 10.32604/cmc.2022.022150 (2022).

[12] Musaed, A., Al-Bawri, S., Islam, M. & Alkadri, W. Parametric analysis of epsilon-negative (ENG) and near zero refractive index (NZRI) characteristics of extraordinary metamaterial for 5G millimetre-wave applications. *IOP Conf. Ser. : Earth Environ. Sci.* **1167**, 012040. 10.1088/1755-1315/1167/1/012040 (2023).

[13] Ram, D., Singh, A. K. & Bhattacharyya, S. A broadband gain-enhanced metasurface-based circularly polarized patch antenna for WLAN application. *Radio Sci.* **60**, 1–13. 10.1029/2024RS008063 (2025).

[14] Ram, D., Singh, A. K. & Bhattacharyya, S. A metasurface-based high gain circularly polarized patch antenna with wide global bandwidth. *J. Electromagn. Waves Appl.* **38**, 1041–1055. 10.1080/09205071.2024.2354718 (2024).

[15] Saleh, C. M. et al. Wideband 5G antenna gain enhancement using a compact single-layer millimeter wave metamaterial lens. *IEEE Access.* **11**, 14928–14942. 10.1109/ACCESS.2023.3244401 (2023).

[16] Babu, K. V. et al. Machine learning-based printed lens antenna with graded-index metasurface for mmWave 5G NR N261 mobile communication applications. *Int. J. Commun. Syst.* **38**, e70176. 10.1002/dac.70176 (2025).

[17] Hussain, N., Jeong, M. J., Abbas, A., Kim, T. J. & Kim, N. A metasurface-based low-profile wideband circularly polarized patch antenna for 5G millimeter-wave systems. *IEEE Access.* **8**, 22127–22135. 10.1109/ACCESS.2020.2969964 (2020).

[18] , P. K. TR A zero-index based metasurface antenna with improved gain and circular polarization characteristics. *Proc. IEEE Tex. Symp. Wirel. Microw. Circuits Syst.* **1-4**10.1109/WMCS52222.2021.9493283 (2021).

[19] Al-Gburi, A. J. A., Ibrahim, I. B. M., Zeain, M. Y. & Zakaria, Z. Compact size and high gain of CPW-fed UWB strawberry artistic shaped printed monopole antennas using FSS single layer reflector. *IEEE Access.* **8**, 92697–92707. 10.1109/ACCESS.2020.2995069 (2020).

[20] Musaed, A. A. et al. High isolation 16-port massive MIMO antenna based negative index metamaterial for 5G mm-wave applications. *Sci. Rep.* **14**, 290. 10.1038/s41598-023-50544-z (2024). 38168653 10.1038/s41598-023-50544-z PMC10762058

[21] Abdelaziem, I. H., Ibrahim, A. A. & Abdalla, M. A. High gain and efficiency dual-band antenna using meta-surface. *AEU - Int. J. Electron. Commun.* **148**, 154163. 10.1016/j.aeue.2022.154163 (2022).

[22] Ali, A., Khalily, M., Brown, T. & Tafazolli, R. Beam-steering capability for OAM-based reflectarray at 5G-mmWave frequencies. *IET Microw. Antennas Propag.* **17**, 162–168. 10.1049/mia2.12330 (2023).

[23] Mao, C., Khalily, M., Xiao, P., Zhang, L. & Tafazolli, R. High-gain phased array antenna with endfire radiation for 26 GHz wide-beam-scanning applications. *IEEE Trans. Antennas Propag.* **69**, 3015–3020. 10.1109/TAP.2020.3024801 (2020).

[24] Balanis, C. A. *Antenna Theory: Analysis and Design* (Wiley, 2015).

[25] Przesmycki, R., Bugaj, M. & Nowosielski, L. Broadband microstrip antenna for 5G wireless systems operating at 28 GHz. *Electronics* **10**, 1. 10.3390/electronics10010001 (2020).

[26] Chen, X., Grzegorczyk, T. M., Wu, B. I., Pacheco, J. & Kong, J. A. Robust method to retrieve the constitutive effective parameters of metamaterials. *Phys. Rev. E*. **70**, 016608. 10.1103/PhysRevE.70.016608 (2004). 10.1103/PhysRevE.70.016608 15324190

[27] Numan, A. B. & Sharawi, M. S. Extraction of material parameters for metamaterials using a full-wave simulator. *IEEE Antennas Propag. Mag*. **55**, 202–211. 10.1109/MAP.2013.6735515 (2013).

[28] Luo, H. et al. Dielectric metamaterials with effective self-duality and full-polarization omnidirectional Brewster effect. *Light Sci. Appl.* **13**, 262. 10.1038/s41377-024-01605-z (2024). 39300089 10.1038/s41377-024-01605-z PMC11412996

[29] Aziz, R. S., Koziel, S. & Pietrenko-Dabrowska, A. Millimeter wave negative refractive index metamaterial antenna array. *Sci. Rep.* **14**, 16037 (2024). 38992148 10.1038/s41598-024-67234-z PMC11239886

[30] Munk, B. A. *Frequency Selective Surfaces: Theory and Design* (Wiley, 2005).

[31] Devarapalli, A. B., Moyra, T. & Mandal, A. Modeling and performance enhancement of compact FSS based low-profile broadband monopole antenna. *Int. J. Commun. Syst.* **37**, e5860. 10.1002/dac.5860 (2024).

[32] Paul, C. R. & Inductance *Loop and Partial* (Wiley, 2011).

[33] Paul, C. R. *Analysis of Multiconductor Transmission Lines* (Wiley, 2007).

[34] Guo, Q., Su, J., Li, Z., Song, J. & Guan, Y. Miniaturized-element frequency-selective rasorber design using characteristic modes analysis. *IEEE Trans. Antennas Propag.* **68**, 6683–6694. 10.1109/TAP.2020.2986640 (2020).

[35] Cao, T. N. et al. Millimeter-wave broadband MIMO antenna using metasurfaces for 5G cellular networks. Int. J. RF Microw. Comput.-Aided Eng. 9938824 (2023). (2023). 10.1155/2023/9938824

[36] Tariq, S., Sethi, W. T., Rahim, A. A., Faisal, F. & Djerafi, T. A metasurface assisted pin loaded antenna for high gain millimeter wave systems. *Sci. Rep.* **15**, 12056. 10.1038/s41598-024-80737-z (2025). 40199878 10.1038/s41598-024-80737-z PMC11978805

[37] Esmail, B. A. & Koziel, S. Design and optimization of metamaterial-based highly-isolated MIMO antenna with high gain and beam tilting ability for 5G millimeter wave applications. *Sci. Rep.* **14**, 3203. 10.1038/s41598-024-53723-8 (2024). 38332321 10.1038/s41598-024-53723-8 PMC10853225

[38] Choi, D. et al. Advanced metamaterial-integrated dipole array antenna for enhanced gain in 5G millimeter-wave bands. *Appl. Sci.* **14**, 9138. 10.3390/app14199138 (2024).

[39] Khan, D., Ahmad, A. & Choi, D. Y. Dual-band 5G MIMO antenna with enhanced coupling reduction using metamaterials. *Sci. Rep.* **14**, 96. 10.1038/s41598-023-50446-0 (2024). 38168470 10.1038/s41598-023-50446-0 PMC10761735

[40] Shrivastava, M. K. et al. A metamaterial loaded high gain low profile RHCP microstrip antenna for X-band applications. *IEEE Access.* **13**, 33321–33329. 10.1109/ACCESS.2025.3542460 (2025).

[41] Esmail, B. A., Isleifson, D. & Koziel, S. Millimeter-wave Yagi MIMO antenna with high isolation and beam-tilting capability using optimized metamaterials. *IEEE Access.* **13**, 107710–107719. 10.1109/ACCESS.2025.3581586 (2025).

